# WIP1 Promotes Homologous Recombination and Modulates Sensitivity to PARP Inhibitors

**DOI:** 10.3390/cells8101258

**Published:** 2019-10-15

**Authors:** Kamila Burdova, Radka Storchova, Matous Palek, Libor Macurek

**Affiliations:** Cancer Cell Biology, Institute of Molecular Genetics of the Czech Academy of Sciences, CZ14220 Prague, Czech Republic; kamila.burdova@img.cas.cz (K.B.); radka.storchova@img.cas.cz (R.S.); matous.palek@img.cas.cz (M.P.)

**Keywords:** DNA repair, phosphatase, genotoxic stress, chemotherapy, PARP inhibitor, olaparib

## Abstract

Genotoxic stress triggers a combined action of DNA repair and cell cycle checkpoint pathways. Protein phosphatase 2C delta (referred to as WIP1) is involved in timely inactivation of DNA damage response by suppressing function of p53 and other targets at chromatin. Here we show that WIP1 promotes DNA repair through homologous recombination. Loss or inhibition of WIP1 delayed disappearance of the ionizing radiation-induced 53BP1 foci in S/G2 cells and promoted cell death. We identify breast cancer associated protein 1 (BRCA1) as interactor and substrate of WIP1 and demonstrate that WIP1 activity is needed for correct dynamics of BRCA1 recruitment to chromatin flanking the DNA lesion. In addition, WIP1 dephosphorylates 53BP1 at Threonine 543 that was previously implicated in mediating interaction with RIF1. Finally, we report that inhibition of WIP1 allowed accumulation of DNA damage in S/G2 cells and increased sensitivity of cancer cells to a poly-(ADP-ribose) polymerase inhibitor olaparib. We propose that inhibition of WIP1 may increase sensitivity of BRCA1-proficient cancer cells to olaparib.

## 1. Introduction

Cells are constantly challenged with DNA damage that comes both from endogenous and exogenous sources. The most deleterious type of DNA damage is double strand breaks (DSBs) that affect both strands of DNA and if not repaired correctly could lead to chromosomal rearrangements. DSBs are repaired by two major pathways—homologous recombination (HR) and non-homologous end joining (NHEJ). NHEJ operates throughout the cell cycle and results in ligation of two ends of DNA that are not extensively processed [1]. HR is restricted to S and G2 phases of the cell cycle as the homologous sequence required as template for repair usually comes from the sister chromatid and the whole process depends on activity of cyclin-dependent kinases [1,2]. Formation of DSBs triggers a highly organized network of protein phosphorylation mediated by PI3-like kinases ATM, ATR and DNA-PK; and ubiquitination mediated by ubiquitin ligases RNF8 and RNF168 [3,4]. DSBs are recognized by either DNA-PK to allow NHEJ or MRE11-RAD50-NBS1 (MRN) complex that starts the process of DNA end resection to allow HR. After initial incision, exonuclease activity of MRE11 removes the DNA towards DSB ends that is followed by long-range resection mediated by Exo1 and DNA2 [2,5]. Resection generates long stretches of single-stranded DNA (ssDNA) that are immediately bound by replication protein A (RPA) that protects it from nucleolytic cleavage [6]. ssDNA-RPA facilitates activation of ATR kinase that further supports repair by HR [7]. In the next step, RPA is exchanged for RAD51 in process mediated by PALB2-BRCA2 that is recruited to sites of damage by BRCA1 or in case of BRCA1 haploinsufficiency by RNF168 [8,9]. RAD51 nucleofilament is stabilized by BRCA1-BARD1 complex and invades the sister chromatid to search for homology that is facilitated by RAD54 [10]. Once homologous sequence is found, DNA is extended using sister chromatid as template. The second end of DNA is eventually captured forming a double holiday junction that is resolved or dissolved yielding either non-crossovers or crossovers.

Two major factors involved in repair pathway choice are TP53-binding protein 1 (53BP1) and Breast cancer type 1 susceptibility protein (BRCA1) [11,12]. BRCA1 association with DSBs is mediated by its interactors RAP80 and BARD1 that mediate binding of the complex to the ubiquitinated histone H2A and histone H4 non-methylated at lysine 20 (H4K20me0), respectively [13,14]. 53BP1 is recruited to DSBs by its BRCT domains that bind phosphorylated histone H2AX (γH2AX), by UDR domain that recognizes histone H2A ubiquitinated at lysine 13/15 by RNF168, and by Tudor domains that recognize dimethylated lysine 20 at histone H4 [14,15,16,17]. After ATM-mediated phosphorylation of 53BP1 on multiple SQ/TQ sites (including Threonine 543), Rap1-interacting factor 1 homolog (RIF1) binds 53BP1 through its Heat repeats and restricts DNA end resection [18,19,20]. Binding of BRCA1 and 53BP1 is not exclusive as in S and G2 phases of the cell cycle BRCA1 can be present at the same DSB as 53BP1. 53BP1 is repositioned from the end of DSB by T543 dephosphorylation mediated by PP4C that is brought to the site by BRCA1 and disrupts interaction between 53BP1 and RIF1 [21]. In addition, BRCA1-BARD1 complex promotes 53BP1 repositioning by ubiquitination of the C-terminal lysines of histone H2A and recruitment of a chromatin remodeler SMARCAD1 [22].

Mutations in BRCA1 and other DNA repair genes are common cause of cancer but deficient HR can be also exploited as target for cancer therapy [23]. Inhibition of poly-(ADP-ribose) polymerase (PARP), a key enzyme in base excision repair, efficiently kills cancer cells with defective HR and PARP inhibitors (including olaparib) are now used for treatment of BRCA1/2-deficient breast and ovary cancer [24]. Combinations of PARPi with other drugs are now being intensively investigated to prevent development of resistance to PARPi and to extend their use beyond the BRCA1/2 negative tumors [24,25,26,27,28,29].

WIP1 is a monomeric magnesium-dependent, chromatin-bound phosphatase encoded by *PPM1D* gene and its expression is increasing towards the G2 phase of the cell cycle [30,31,32]. WIP1 terminates the DNA damage response by dephosphorylation of γH2AX, ATM pS1981 and KAP1 pS824 and promotes release from the cell cycle checkpoint by dephosphorylation of p53 pS15 [30,33,34,35,36,37]. *PPM1D* locus is amplified in about 10% of breast cancers, in medulloblastoma and ovary cancer [38,39,40]. Importantly, *PPM1D* amplifications occur mostly in tumors harboring wild-type p53 [38,41]. Activity of WIP1 can be specifically inhibited by a small-molecule compound GSK2830371 and WIP1 was proposed as perspective pharmacological target particularly in p53-proficient cancers [42,43,44,45,46].

Here we report a novel role of WIP1 in DSB repair through HR. We find that WIP1 stably interacts with BRCA1-BARD1 complex and inhibition of WIP1 delays recruitment of BRCA1 to DSBs. Consistent with WIP1 function in HR, inhibition of WIP1 leads to accumulation of DNA damage in S/G2 cells and sensitizes cancer cells to olaparib. Thus, inhibition of WIP1 may promote efficiency of PARP inhibitors in tumors with normal BRCA1 function.

## 2. Results 

### 2.1. WIP1 Promotes DSB Repair by Homologous Recombination

**Figure 1:**
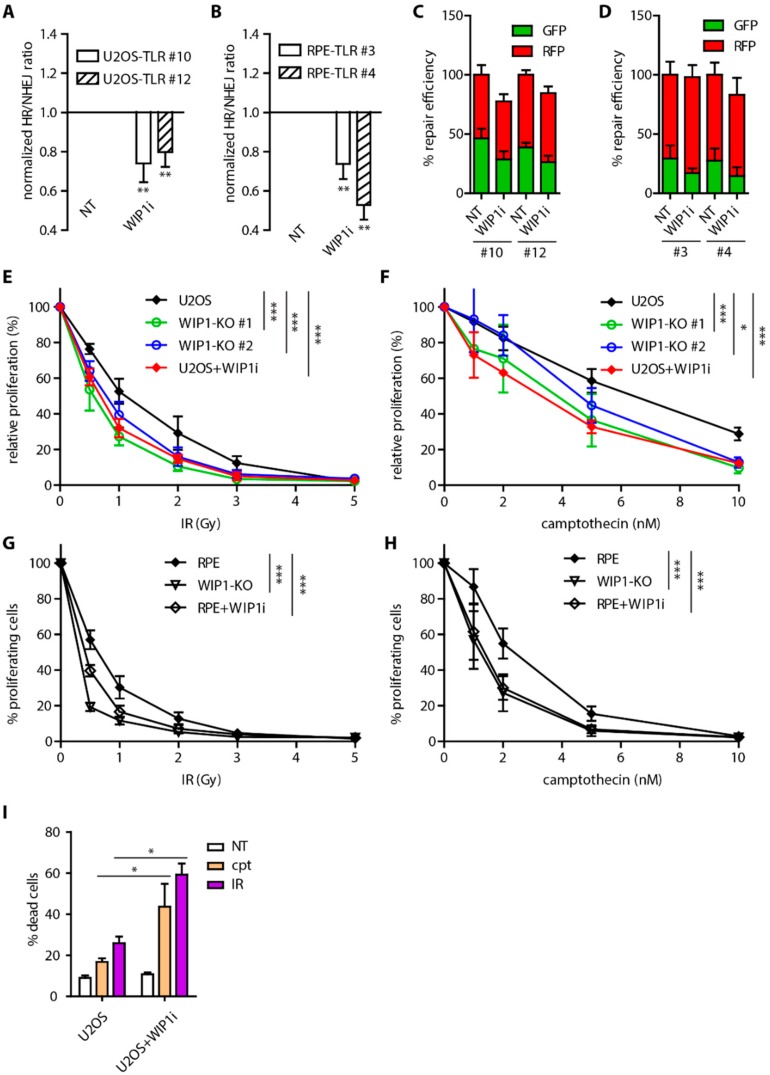
Inhibition of WIP1 impairs homologous recombination (HR). (**A**) Traffic light reporter assay in U2OS cells. Two independent stable cell lines (clones #10 and #12) were transfected with ISceI together with BFP-donor vector with or without pretreatment with 1 μM WIP1i. Efficiency of repair was analyzed 3 days after transfection by FACS. Plotted is mean of normalized ratio of GFP^+^/RFP^+^ cells. Bars indicate SD, n ≥ 3. Statistical significance evaluated by two-tailed *t*-test. (**B**) Traffic light reporter assay in two independent clones of RPE cells (#3 and #4). Same as A. (**C**) Efficiency of repair by HR (GFP^+^) and NHEJ (RFP^+^) in TLR assay in U2OS cells from A. (**D**) Efficiency of repair by HR (GFP^+^) and NHEJ (RFP^+^) in TLR assay in RPE cells from B. (**E**) Cell survival after irradiation of parental U2OS and two independent U2OS-WIP1-KO cell lines treated or not with WIP1 inhibitor was evaluated after 7 days using resazurin viability assay. Plotted is mean and SD, n ≥ 3. Statistical significance evaluated by two-way ANOVA (* *P* < 0.05; *** *P* < 0.001). (**F**) Cell survival of parental U2OS and two independent U2OS-WIP1-KO cell lines treated with indicated doses of camptothecin with or without combined treatment with WIP1 inhibitor was evaluated after 7 days using resazurin viability assay. Plotted is mean and SD, n ≥ 3. Statistical significance evaluated by two-way ANOVA (* *P* < 0.05; *** *P* < 0.001). (**G**) Cell survival after irradiation of parental RPE and RPE-WIP1-KO cell lines assayed as in E. (**H**) Cell survival of parental RPE and RPE-WIP1-KO cell lines with treated with camptothecin and analyzed as in F. (**I**) Percentage of dead cells was evaluated by Hoechst 33258 staining and FACS analysis 7 days after treatment with camptothecin or after irradiation in U2OS cell line with or without combined treatment with WIP1i. Plotted is mean +/− SD. Statistical significance evaluated by two-tailed *t*-test.

WIP1 phosphatase was shown to counteract ATM kinase activity at chromatin to terminate DNA damage response and to facilitate recovery form the G2 checkpoint [30,34,35]. In addition, overexpression of WIP1 affects DSB repair efficiency through dephosphorylation of γH2AX leading to disruption of DDR signaling [30,47]. To evaluate the role of WIP1 in more physiological condition we used different established cell based reporter assays together with a recently described specific WIP1 inhibitor GSK2830371 [42,44]. To this end we generated stable Traffic light reporter cell lines in U2OS and RPE that allowed us to analyze the overall repair efficiency as well as the ratio of repair efficiency by homologous recombination (GFP+) and non-homologous end joining (RFP+) (Figure S1A) [48]. As expected, inhibition of DNA-PK increased the HR/NHEJ ratio reflecting its essential role in NHEJ (Figure S1B). Conversely, inhibition of ATM decreased the HR/NHEJ ratio which is consistent with involvement of ATM in mediating DNA resection (Figure S1B) [49]. Interestingly, inhibition of WIP1 lowered DSB repair efficiency by homologous recombination while NHEJ was not affected and thus decreased the HR/NHEJ ratio in two independent clones of both U2OS and RPE cells (Figure 1A–D). To further confirm this phenotype, we used established U2OS DR-GFP and E5J reporter cell lines and consistently we observed decreased HR efficiency after inhibition of WIP1 (Figure S1C) [50].

Next, we employed CRISPR/Cas9 technology and generated WIP1 knockout U2OS and RPE cell lines (Figure S1D,E) and tested their sensitivity to gamma irradiation (IR). As reported previously, U2OS-WIP1 knockout cells were more sensitive to IR and their sensitivity was comparable to WIP1 inhibition (Figure 1E,G) [44]. Importantly, increased sensitivity was partially rescued by complementation of knockout cells with wild-type WIP1 but not catalytically inactive mutant D314A (Figure S1F,G). Treatment of cells by topoisomerase I inhibitor camptothecin was shown to induce DNA damage that is repaired by HR [51]. Both RPE and U2OS WIP1 knockout cell lines were found to be more sensitive to camptothecin treatment to similar extent as after WIP1 inhibition (Figure 1F,H). Decreased cell proliferation after inhibition of WIP1 was accompanied by increased cell death after IR or camptothecin treatment (Figure 1I). Consistent with potential role of WIP1 in HR, U2OS-WIP1-KO cells were more sensitive to DNA crosslinking agent mitomycin C (Figure S1H).

**Figure 2:**
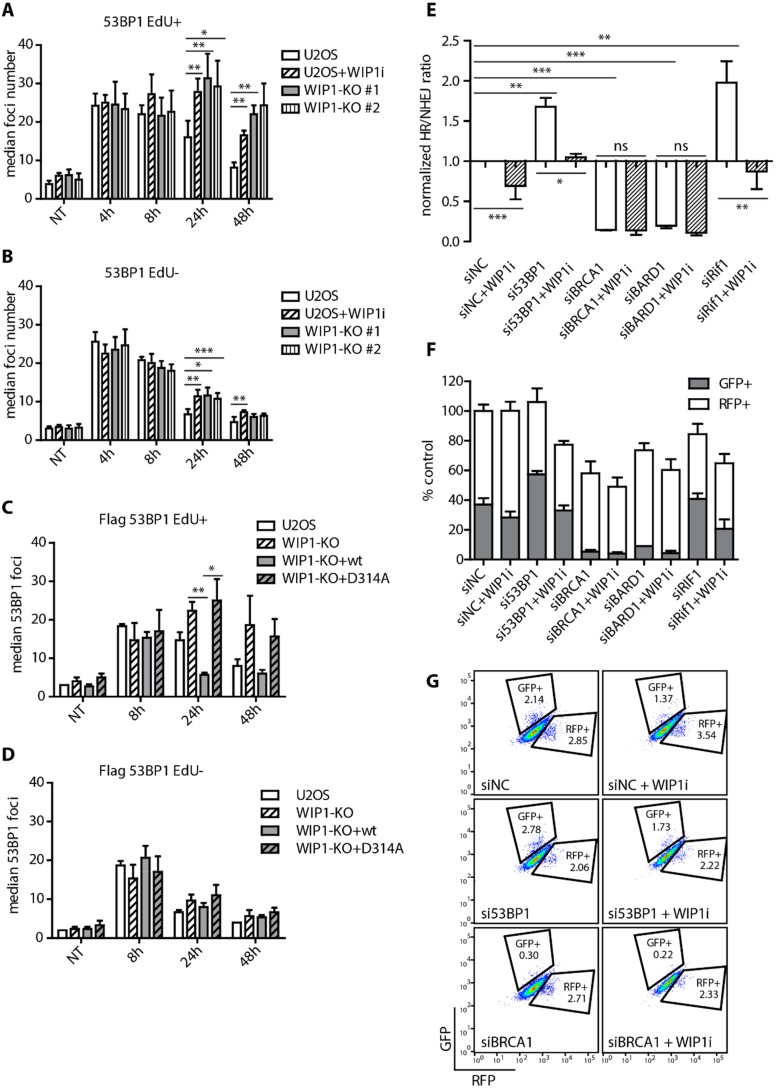
WIP1 plays role in DNA double-strand break repair in S-phase cells. (**A**) Quantification of 53BP1 foci in replicating (EdU+) cells after irradiation. U2OS parental cell lines with or without combined treatment with WIP1i and two independent WIP1 knockout cell lines were pulse-labeled with EdU for 30 min before irradiation. Cells were fixed after pre-extraction at indicated time-points and stained with 53BP1 antibody. Click chemistry was used to visualize EdU. Mean of median foci number +/- SD is plotted (n ≥ 3). Statistical significance evaluated by two tailed *t*-test. (**B**) Quantification of 53BP1 foci in non-replicating (EdU-) cells after irradiation. As in A. (**C**) Quantification of 53BP1 foci in replicating (EdU+) cells after irradiation. U2OS parental, WIP1 knockout and cell lines complemented with wild-type or phosphatase-dead (D314A) mutant of WIP1 were irradiated and analyzed as in A. (**D**) Quantification of 53BP1 foci in non-replicating (EdU-) cells after irradiation. U2OS parental, WIP1 knockout and cell lines complemented with wild-type or phosphatase-dead (D314A) mutant of WIP1 were irradiated and analyzed as in A. (**E**) Traffic light reporter assay in U2OS cells after transfection with indicated siRNA. Cells were transfected with ISceI together with BFP-donor vector with or without pretreatment with 1 μM WIP1i 2 days after siRNA transfection. Efficiency of repair was analyzed by FACS 3 days after ISceI and BFP-donor transfection. Plotted is mean +/− SD. Statistical significance evaluated by two-tailed *t*-test. (**F**) Efficiency of repair by HR and NHEJ in Traffic light reporter assay as in E. (**G**) Representative plots from Traffic light reporter assay in E.

Next, we followed DSB repair kinetics in parental U2OS or U2OS-WIP1-KO cells by quantifying 53BP1 foci formation and disassembly after exposure to IR. Interestingly, knockout or inhibition of WIP1 lead to persistence of 53BP1 foci mainly in cells that were in S-phase (EdU+, Figure 2A, and Figure S2A) at time of irradiation and to lesser extent in cells irradiated in G1 or G2 phases of the cell cycle (EdU-, Figure 2B and Figure S2A). Persistence of 53BP1 foci was fully rescued in WIP1 knockout cells complemented with the wild-type WIP1 but not with D314A mutant (Figure 2C,D and Figure S2B). Moreover, persistence of 53BP1 foci in cells irradiated in S-phase was recapitulated in MCF7 cells treated with WIP1 inhibitor (Figure S3A–C).

DNA repair pathway choice is controlled by a balance between 53BP1 and BRCA1 at DNA double strand breaks that have opposing effects on DNA end resection [11,19]. To evaluate possible impact of WIP1 on these proteins, we employed the Traffic light reporter assay and depleted 53BP1/RIF1 and/or BRCA1/BARD1 using siRNA in combination with WIP1 inhibition. As expected, depletion of BRCA1 or BARD1 decreased HR frequency, whereas depletion of 53BP1 or RIF1 increased the HR/NHEJ ratio [19,48,52,53]. Importantly, HR was not further decreased upon WIP1 inhibition in BRCA1 and BARD1-depleted cells (Figure 2E–G and Figure S1I). In contrast, increased HR observed in 53BP1 and RIF1-depleted cells was reduced back to normal after inhibition of WIP1 (Figure 2E–G). Combined these data suggest that WIP1 may promote HR through regulation of BRCA1/BARD1 complex.

### 2.2. WIP1 Interacts with BRCA1 and Promotes its Recruitment to DSBs

**Figure 3:**
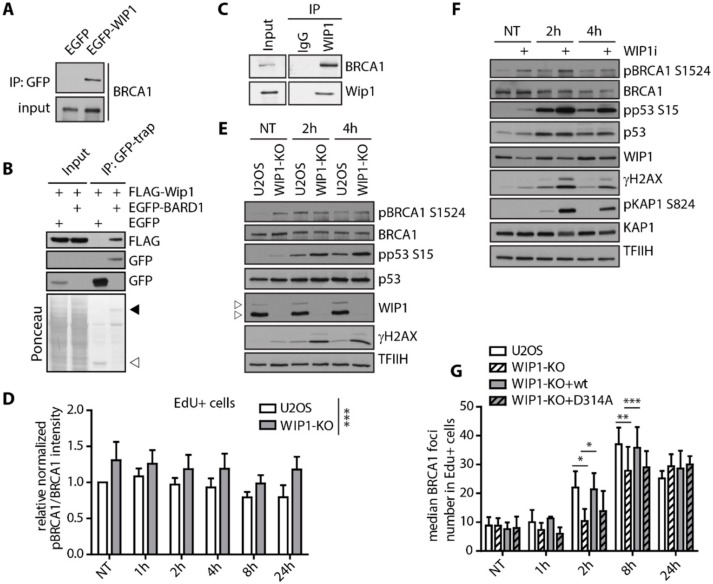
WIP1 interacts with BRCA1 and dephosphorylates S1524. (**A**) Co-immunoprecipitation of WIP1 and BRCA1. HEK293 cells were transfected with either empty GFP or GFP-WIP1, subjected to immunoprecipitation using GFP-Trap and analyzed by Western blotting with BRCA1 antibody. (**B**) Co-immunoprecipitation of WIP1 and BARD1. HEK293 cells were co-transfected with either empty GFP or GFP-BARD1 and Flag-WIP1, subjected to immunoprecipitation using GFP-Trap and analyzed by Western blotting with indicated antibodies. Ponceau staining with indicated positions of GFP (empty arrowhead) and GFP-BARD1 (full arrowhead) are shown. (**C**) Co-immunoprecipitation of endogenous WIP1 and BRCA1. U2OS cell lysates were incubated with 2 μg of a control antibody (IgG) or affinity-purified antibody against WIP1 for 2 h. Protein complexes were isolated by protein A/G resin and analyzed by immunoblotting. (**D**) Quantification of BRCA1 pS1524 signal intensity in replicating (EdU+) cells after irradiation. U2OS parental and WIP1 knockout cell lines were pulse-labeled with EdU for 30 min before irradiation. At indicated time-points, cells were pre-extracted, fixed and stained with pBRCA1 S1524 and BRCA1 antibodies. Click chemistry was used to visualize EdU. Median total intensity of BRCA1 pS1524 was normalized to total BRCA1 and is plotted +/− SD. Statistical significance evaluated by two-way ANOVA. (**E**) Western blot analysis of U2OS parental and U2OS-WIP1-KO cell lines after irradiation. Cells were irradiated and whole cell lysates were analyzed using Western blotting with indicated antibodies. Arrowheads indicate two isoforms of WIP1 present in U2OS. (**F**) Western blot analysis of MCF7 cells after irradiation with or without combined treatment with WIP1i. Cells were pretreated with WIP1 inhibitor for 30 min before irradiation and whole cell lysates were analyzed by Western blotting with indicated antibodies. (**G**) Quantification of BRCA1 foci in replicating (EdU+) cells after irradiation. Parental U2OS and U2OS-WIP1-KO cells and cell lines complemented with wild-type or phosphatase-dead (D314A) mutant of WIP1 were pulse-labeled with EdU for 30 min before irradiation. Cells were fixed after pre-extraction at indicated time-points and stained with BRCA1 antibody. Click chemistry was used to visualize EdU. Mean of median total intensity +/− SD is plotted. Statistical significance evaluated by two-tailed *t*-test.

To investigate the impact of WIP1 on BRCA1, we first performed a set of immunoprecipitation assays. We observed that WIP1 co-immunoprecipitated with BRCA1 and BARD1 in non-treated HEK293 and U2OS cells suggesting that WIP1 forms a stable interaction with BRCA1-BARD1 complex (Figure 3A–C). BRCA1 and BARD1 were previously reported to be extensively phosphorylated by ATM/ATR after DNA damage [54,55,56,57,58]. To determine BRCA1 phosphorylation after DNA damage we validated the phosphospecific BRCA1-pS1524 antibody for both immunofluorescence and Western blotting (Figure S4A–D). As expected, total intensity of BRCA1-pS1524 at chromatin was increased in response to IR in both S and G2 cells whereas RNAi-mediated depletion of BRCA1 reduced the signal to the basal level (Figure S4A,B). Next, we performed in vitro phosphatase assay and established that recombinant His-WIP1 was able to dephosphorylate BRCA1 S1524 with a comparable efficiency to other substrates including ATM S1981, KAP1 S824 and p53 S15 (Figure S4E). In addition, purified WIP1 dephosphorylated BRCA1 S1524 in the presence of ATM inhibitor and also in fixed cells indicating that removal of the signal was not caused by modulation of ATM activity (Figure S4J,K). Interestingly, basal BRCA1 phosphorylation at S1524 was increased in WIP1 knockout cells and there was no further increase in BRCA1-pS1524 signal after IR compared to untreated condition (Figure 3D,E and Figure S5A). Similar effect was observed in MCF7 cells treated with WIP1 inhibitor confirming that WIP1 dephosphorylates BRCA1 not only after IR but also in unchallenged conditions (Figure 3F). Next, we assayed the recruitment of BRCA1 to the foci formed in S phase cells after exposure to IR. We observed delayed formation of BRCA1 foci in early time-points in WIP1 knockout cell line that could be rescued by complementation with the wild-type WIP1 but not with inactive D314A mutant (Figure 3G and Figure S5B). Combined these data indicate that WIP1 forms a stable complex with BRCA1-BARD1 and its activity is needed for timely recruitment of BRCA1 to DSBs.

### 2.3. WIP1 Dephosphorylates 53BP1 at T543 Residue Needed for Interaction with RIF1

**Figure 4:**
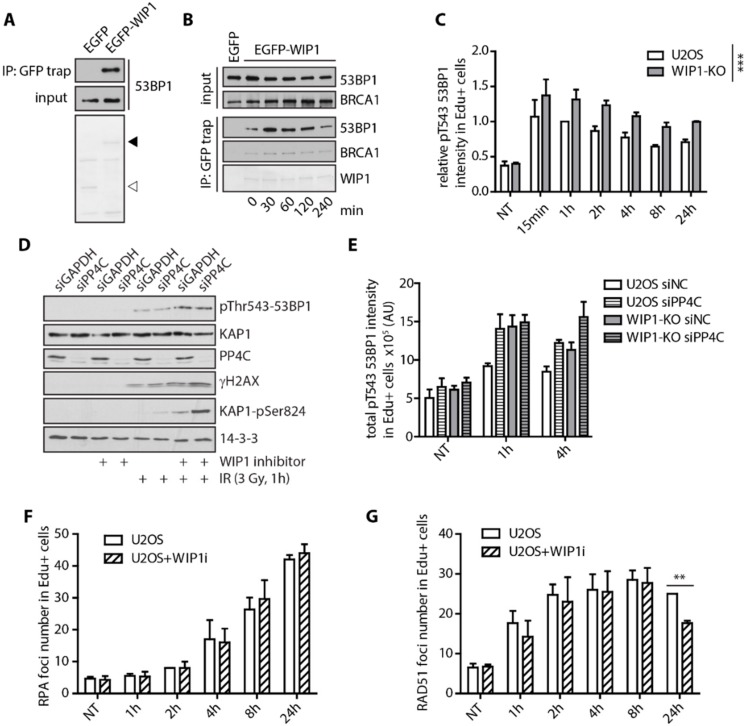
WIP1 delays recruitment of BRCA1 and dephosphorylation of 53BP1 at T543. (**A**) Co-immunoprecipitation of WIP1 and 53BP1. HEK293 cells were transfected with either empty GFP or GFP-WIP1, subjected to immunoprecipitation using GFP-Trap 24 h after transfection and by Western blotting with 53BP1 antibody. Ponceau staining with indicated positions of GFP (empty arrowhead) and GFP-WIP1 (full arrowhead) are shown. (**B**) HEK293 cells transfected with EGFP or EGFP-WIP1 were exposed to 3 Gy of IR, collected at indicated times and proteins were immunoprecipitated by GFP Trap. (**C**) Quantification of 53BP1 pT543 signal intensity in replicating (EdU+) cells after irradiation. U2OS parental and WIP1 knockout cell lines were pulse-labeled with EdU for 30 min before irradiation. Cells were fixed after pre-extraction at indicated time-points after IR and stained with p53BP1 T543 antibody. Click chemistry was used to visualize EdU. Mean of median total intensity +/− SD is plotted. (**D**) Western blot analysis of whole cell lysates of U2OS cells transfected with GAPDH or PP4C siRNA in response to irradiation and/or WIP1 inhibitor. (**E**) Quantification of 53BP1 pT543 signal intensity in replicating (EdU+) cells after irradiation. U2OS parental and WIP1 knockout cell lines were transfected with control or PP4C siRNA 2 days before irradiation. Cells were processed and analyzed as in C. (**F**) Quantification of RPA2 foci in replicating (EdU+) cells after irradiation. U2OS parental cell lines with or without combined treatment with WIP1i were pulse-labeled with EdU for 30 minutes before irradiation. Cells were fixed after pre-extraction at indicated time-points and stained with RPA2 and RAD51 antibodies. Click chemistry was used to visualize EdU. Mean of median foci number +/− SD is plotted. (**G**) Quantification of RAD51 foci in replicating (EdU+) cells after irradiation as in F.

Next, we aimed to test possible impact of WIP1 on 53BP1. Using immunoprecipitation, we found that WIP1 interacted with 53BP1 (Figure 4A). However, in contrast to the stable interaction with BRCA1, we observed increased interaction between WIP1 and 53BP1 after exposure to ionizing radiation (Figure 4B). BRCA1 was recently implicated in 53BP1 repositioning after IR by mediating 53BP1 dephosphorylation at threonine 543 and releasing its interaction with RIF1 [21]. Using siRNA of 53BP1 we validated the specificity of the pT543 53BP1 antibody for immunofluorescence and Western Blotting (Figure S4F–H). In addition, we found that WIP1 efficiently dephosphorylated 53BP1 at T543 in vitro (Figure S4I–K). Whereas PP4C was originally reported to mediate pT543 dephosphorylation [21], we noted a significant increase of 53BP1 phosphorylation at T543 in WIP1 knockout cell line in response to IR by immunofluorescence and in cells treated with WIP1 inhibitor by immunoblotting (Figure 4C,D and Figure S6). Partial overlap in substrate specificity between PP4C and WIP1 has previously been reported for other substrates including γH2AX and KAP1 [30,35,59,60] and similarly both phosphatases may collaborate to control the phosphorylation status of 53BP1. Indeed, at later time-points after IR we observed a more pronounced 53BP1 T543 phosphorylation after combining depletion of PP4C and knockout of WIP1 (Figure 4E).

As WIP1 interacts with and dephosphorylates BRCA1 and 53BP1, we aimed to evaluate its role in DNA resection that is controlled by the balance between BRCA1 and 53BP1 at DSBs. Surprisingly, we did not observe any difference in formation of RPA2 foci in S-phase cells after inhibition of WIP1 (Figure 4F). Similarly, formation of RAD51 filament was largely unaffected in early time-points after irradiation (Figure 4G) suggesting that WIP1 does not influence DNA end resection.

### 2.4. WIP1 Deficient Cells are Sensitive to PARP Inhibition in BRCA1 Dependent Manner

**Figure 5:**
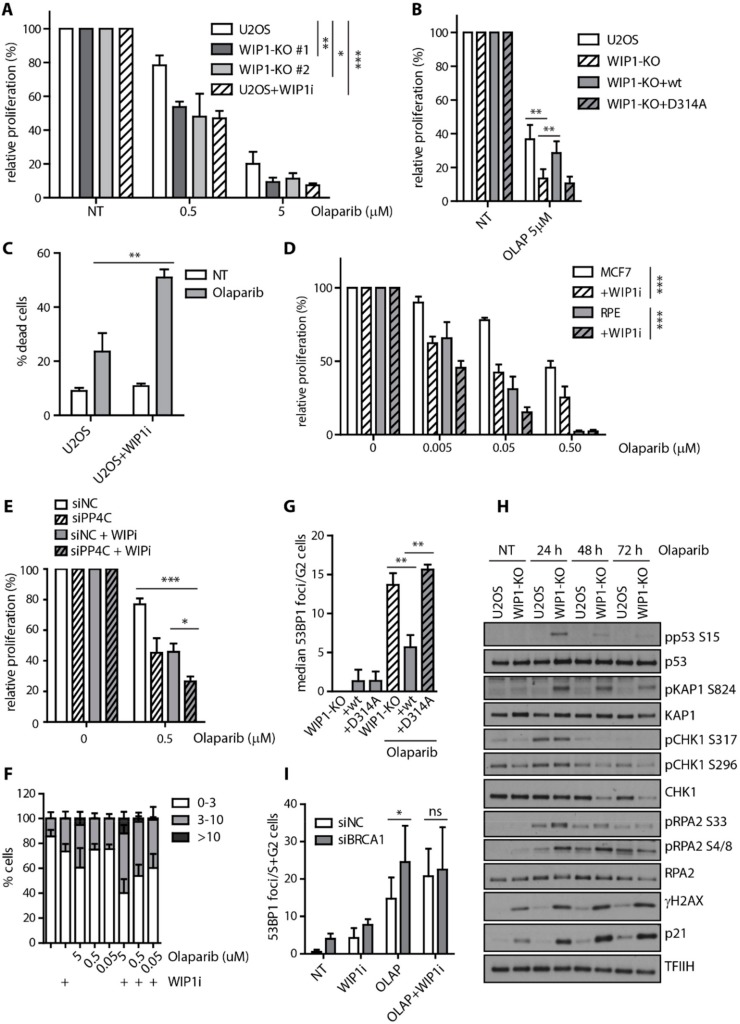
WIP1 deficient cells are more sensitive to PARP inhibition. (**A**) Cell survival of parental U2OS, two independent U2OS-WIP1-KO cell lines with or without combined treatment with WIP1i was evaluated 7 days after treatment with indicated doses of olaparib using resazurin viability assay. Plotted is mean +/− SD, n ≥ 3. Statistical significance evaluated by two-way ANOVA. (**B**) Cell survival of parental U2OS, U2OS-WIP1-KO cells and cell lines complemented with wild-type or phosphatase-dead (D314A) mutant of WIP1 in response to 5 μM olaparib as in A. Statistical significance evaluated by two-tailed *t*-test (n ≥ 3). (**C**) Percentage of dead cells was evaluated by Hoechst 33258 staining and FACS analysis 7 days after treatment with 5 μM olaparib in U2OS cell line with or without combined treatment with WIP1i. Plotted is mean +/− SD. (**D**) Cell survival of RPE and MCF7 cell lines with or without combined treatment with WIP1i was evaluated 7 days after treatment with indicated doses of olaparib using resazurin viability assay. Plotted is mean +/− SD. N ≥ 3. Statistical significance evaluated by two-way ANOVA. (**E**) Cells were transfected with control siRNA (siNC) or siRNA to PP4C (siPP4C). Cell survival was evaluated after 7 days of treatment with olaparib and DMSO or WIP inhibitor. Statistical significance evaluated by two-tailed *t*-test (n = 3). (**F**) Quantification of 53BP1 foci number 3 days after treatment with olaparib. U2OS cells were treated with indicated doses of olaparib together with or without WIP1i for 3 days, fixed, stained with 53BP1 antibody and percentage of cells having 0–3, 3–10 and >10 foci were quantified. Mean +/− SD is plotted, n ≥ 3. (**G**) Quantification of 53BP1 foci after treatment with olaparib. U2OS-WIP1-KO cells and cell lines complemented with wild-type or phosphatase-dead (D314A) mutant of WIP1 were treated with WIP1i and olaparib for 3 days, fixed after pre-extraction and stained with 53BP1 antibody. Number of 53BP1 foci in S/G2 cells was evaluated using DAPI content of >2 n to gate S-G2 cells. Mean of median foci number +/− SD is plotted, n ≥ 3. Statistical significance evaluated by two-tailed *t*-test. (**H**) Response of U2OS and U2OS-WIP1-KO cell lines to treatment with 5 μM olaparib for 24–72h was analyzed by Western blotting using indicated antibodies. **I**) Quantification of 53BP1 foci 3 days after treatment with olaparib. MCF7 cells were transfected with indicated siRNAs and treated after 2 days with WIP1i and olaparib alone or combined for further 3 days. Cells were fixed after pre-extraction and stained with 53BP1 antibody. Number of 53BP1 foci in S/G2 cells was evaluated using DAPI content of >2 n to gate S-G2 cells. Mean of median foci number +/− SD is plotted. Statistical significance evaluated by two-tailed *t*-test.

Mutations in BRCA1/2 that impair HR are commonly found in breast and ovarian cancers and increase sensitivity to PARP inhibitors. Since inhibition of WIP1 impaired HR, we tested if WIP1 deficiency would lead to sensitization of cells to PARP inhibitors. Indeed, we found that U2OS WIP1 knockout cell lines were more sensitive to olaparib (Figure 5A). Importantly, WIP1 inhibition decreased cell proliferation to the similar extent as the knockout cell lines (Figure 5A) and loss of WIP1 could be rescued by complementation with the wild-type WIP1 but not catalytically inactive D314A mutant (Figure 5B). Decreased cell proliferation after combined treatment with WIP1 inhibitor and olaparib was associated with increased cell death in U2OS cells (Figure 5C). Moreover, similar increase in sensitivity to olaparib and another PARPi A-966492 [61] was observed after inhibition of WIP1 in MCF7 and RPE cell lines (Figure 5D, Figure S7A). Combined depletion of PP4C and inhibition of WIP1 further increased sensitivity of cells to olaparib, suggesting that both phosphatases may target similar substrates involved in regulation of HR (Figure 5E).

WIP1 inhibition increased the number of 53BP1 foci in U2OS and MCF7 cells in response to PARP inhibition (Figure 5F, Figure S7B,C) and was accompanied by increased γH2AX intensity suggesting that DNA lesions accumulate after the combined treatment (Figure S7D,E). The increase of 53BP1 foci number was observed mainly in S/G2 phases of the cell cycle which is consistent with accumulation of DNA damage due to failed HR (Figure S7F,G). Importantly, accumulation of 53BP1 foci in S/G2 cells was rescued by complementation with the wild-type WIP1 but not catalytically inactive D314A mutant (Figure 5G).

Next, we analyzed the response of U2OS and WIP1 knockout cells to olaparib treatment. As expected, treatment of U2OS cells with olaparib induced CHK1 and RPA2 phosphorylation after 24 h and was followed by a slight increase of γH2AX and p21 levels after 2–3 days (Figure 5H). In contrast, treatment of U2OS WIP1 knockout cells with olaparib lead to additional increase of H2AX and RPA2 phosphorylation accompanied by a strong induction of p21 protein levels which is consistent with an increased load of DNA damage leading to a profound activation of the cell cycle checkpoint (Figure 5H). Indeed, we found that cells treated with a combination of olaparib and WIP1 inhibitor accumulated in the G2 (Figure S7F). Cells that entered mitosis in the presence of olaparib and WIP1 inhibitor showed increased frequency of abnormal anaphases likely reflecting a presence of unrepaired DNA (data not shown). To test if the sensitivity of U2OS WIP1 knockout cells to PARP inhibitors is due to the increased activation of the cell cycle checkpoint mediated by p21, we generated p21 knockout cell line (Figure S7H). Interestingly, the effect of WIP1 inhibition was found to be p21 independent in cell survival assays (Figure S7I). Moreover, inhibition of WIP1 further increased the number of 53BP1 foci induced by olaparib in p21 deficient U2OS cells (Figure S7J). We conclude that the increased sensitivity of cells observed after combined treatment with WIP1 and PARP inhibitors is not caused by a stronger activation of the cell cycle checkpoint caused by inhibition of WIP1. To test whether the increased load of DNA damage after combined inhibition of PARP and WIP1 was BRCA1 dependent, we depleted MCF7 cells of BRCA1 using siRNA and analyzed number of 53BP1 foci in S/G2 phases of cell cycle. Indeed, we found that depletion of BRCA1 is epistatic with inhibition of WIP1 after olaparib treatment (Figure 5I).

## 3. Discussion

WIP1 phosphatase prevents induction of senescence in cells exposed to genotoxic stress and promotes recovery from the G2 checkpoint through targeting the p53 pathway and a nuclear co-repressor KAP1 [35,36,62]. In addition to this established role in checkpoint silencing, WIP1 was also reported to impact on the nucleotide and base excision repair pathways by targeting XPA and UNG2 at chromatin [63,64]. Here we identified a novel role of WIP1 in promoting repair of DSBs through HR in S/G2 cells. Using two distinct reporter assays we showed that HR (but not NHEJ) efficiency was decreased upon inhibition of WIP1. This was accompanied by a delayed clearance of the 53BP1 foci indicating the persistent DNA damage in S/G2 cells lacking WIP1. We found that WIP1 interacted with and dephosphorylated BRCA1 whereas loss of WIP1 delayed recruitment of BRCA1 to the DSBs. Loss of WIP1 delayed dephosphorylation of 53BP1 at a residue previously reported to mediate interaction with RIF1 and promote chromatin remodeling. Although we observed a higher impact on T543 53BP1 phosphorylation by inhibiting WIP1 than by depletion of PP4C that was previously reported to target 53BP1, we failed to detect any significant difference in the DNA resection upon inhibition of WIP1. It is plausible that WIP1 affects 53BP1 repositioning only in a small but physiologically meaningful fraction of DNA lesions depending on the context of the chromatin. Alternatively, WIP1 may fine-tune HR through additional substrates involved in the late steps of HR. One of the candidates is the BRCA1-BARD1 complex that is phosphorylated at multiple sites by ATM/ATR, stably interacts with WIP1 and was recently shown to be important for invasion step of HR [10]. Accumulation of 53BP1 foci in G2 cells caused by a combined treatment with olaparib and WIP1 inhibitor was independent on the ability of cells to activate the cell cycle checkpoint. However, we cannot exclude that WIP1 modulates HR also through inhibition of p53 that was previously shown to directly interact with RAD51 and RAD54 and to suppress RAD51 expression [65,66,67]. The precise molecular mechanism of WIP1 function in HR will need to be addressed by future research.

**Figure 6:**
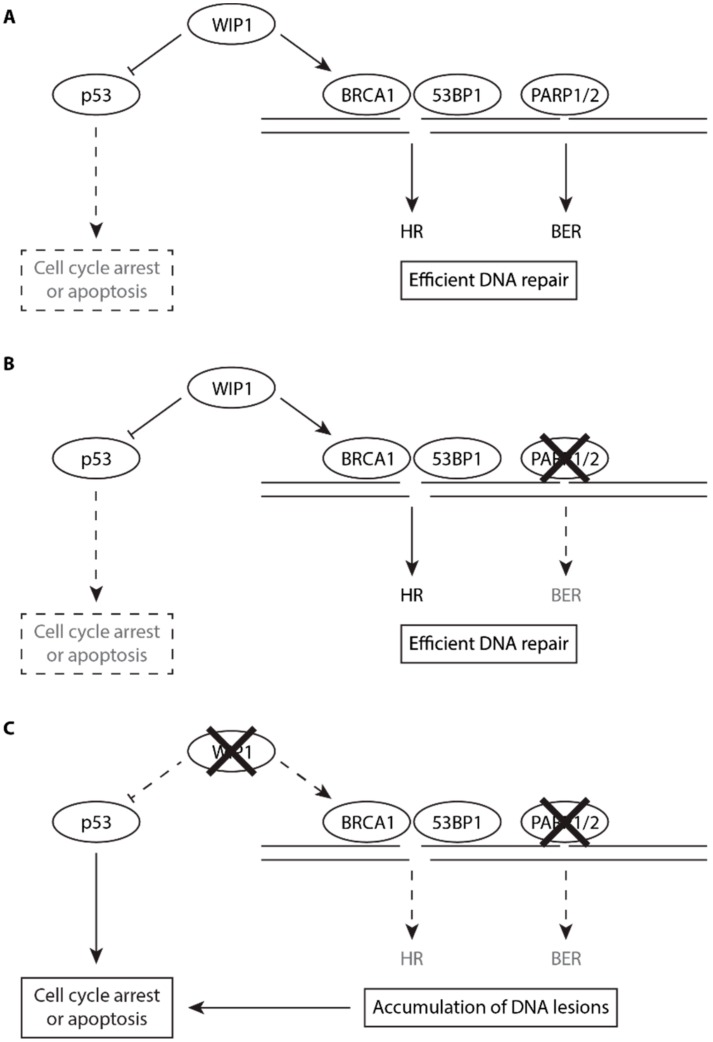
Putative model for the role of WIP1 in HR and in PARP inhibitor sensitivity. (**A**) Under normal conditions, endogenous DNA lesions are efficiently repaired by HR and BER. WIP1 promotes efficiency of HR and limits the extent of p53 pathway activation allowing cells to proliferate. (**B**) After inhibition of PARP1, BER pathway is impaired (dashed lines) but DNA lesions are efficiently repaired by HR. (**C**) Combined inhibition of PARP1 and WIP1 impairs both BER and HR (dashed lines) and enhances p53 response leading to accumulation of DNA lesions in G2 cells. Increased DNA damage load triggers the DNA damage response and allows full activation of p53 pathway leading to cell death.

PARP inhibitors are currently approved for treatment of BRCA1/2-deficient tumors and new drug combinations are under investigation. Consistent with the WIP1 role in HR, we observed that loss of WIP1 promoted sensitivity of cancer cells to PARP inhibitors. Combined treatment with olaparib and WIP1 inhibitor increased the DNA damage load in G2 cells and significantly increased cell death. In contrast to other phosphatases, WIP1 activity can be specifically suppressed by a specific inhibitor GSK2830371 and WIP1 inhibition is well tolerated in normal cells. Based on the newly identified role of WIP1 in HR, we propose WIP1 phosphatase as potential pharmacological target in BRCA1-proficient tumors (Figure 6). Inhibition of WIP1 was previously reported to be efficient mainly in p53-proficient cancer types including neuroblastoma, breast adenocarcinoma and melanoma [44,68,69,70]. We hypothesize that inhibition of HR and stimulation of the p53 response could synergize to eradicate the cancer cells treated with WIP1 inhibitors.

## 4. Material and Methods

### 4.1. Cell Lines

All cell lines used were maintained in DMEM containing 6% FBS, Penicillin (100 U/mL) and Streptomycin (0.1 mg/mL). All cell lines were regularly checked for mycoplasma contamination and were confirmed as negative. U2OS-WIP1-KO cells were described previously [44] and here were stably complemented by transfection of EGFP-WIP1-wt or EGFP-WIP1-D314A followed by three weeks selection by zeocin and expansion of individual GFP+ clones. WIP1 knockout in RPE cells was generated by transfection of pCMV-CAS9-2A-GFP (Sigma-Aldrich, St. Louis, MO, USA) carrying gRNA sequence tgagcgtcttctccgaccaggg, followed by sorting of single GFP+ cells 48 h after transfection to 96-well plate. Loss of WIP1 was validated by Western blotting in single clones. Knockout of *CDKN1A/p21* in U2OS cells was generated using CRISPR-Cas9 and HDR reporter vector (Santa Cruz Biotechnology, Dallas, TX, USA) as described [44]. Cells were sorted as GFP+/RFP+ 48 h after plasmid transfection as single cells to 96-well plate and knockout was validated by Western blotting in single clones. Traffic light reporter cell lines were generated by transfection of linearized pCVL Traffic Light Reporter 1.1 Ef1a Puro plasmid (Addgene, Watertown, MA, USA, Plasmid #31482) [48] to U2OS or RPE cells using polyethylenimine. Single clones were picked after selection with puromycin for three weeks. Integration of the reporter was confirmed using ISceI with BFP-donor plasmid transfection by FACS. Silencer Select siRNA was transfected at 5 nM final concentration using RNAiMAX using manufacturer’s guidelines (Thermo Fisher Scientific, Waltham, MA, USA). Where indicated, cells grown on culture plates were exposed to the indicated dose of ionizing radiation generated by X-RAD 225XL instrument with Cu filter 0.5 mm (Precision X-Ray, North Branford, CT, USA).

### 4.2. Antibodies and Chemicals

Following antibodies were used: WIP1 antibody (clone F-10, sc-376257), p21 (sc-397), p53 (clone D01, sc-126), BRCA1 (sc-6954), rabbit-53BP1 (sc-22760), RAD51 (sc-6862) and TFIIH (sc-293, used as loading control) from Santa Cruz Biotechnology (Dallas, TX, USA); phoshpo-Thr543-53BP1 (#3428), phospho-S15-p53 (#9284) from Cell Signaling Technology (Danvers, MA, USA); RPA2 (clone 9H8, ab2175), and phospho-Ser1524-BRCA1 (ab2401) from Abcam (Cambridge, UK), γH2AX (05-636), and mouse monoclonal 53BP1 (MAB3802) from Merck Millipore (Burlington, MA, USA); phospho-S824-KAP1 (GTX63711), KAP1 (GTX62973) and PP4C (GTX114659) from Genetex (Irvine, CA, USA); secondary Alexa Fluor conjugated antibodies from Thermo Fisher Scientific (Waltham, MA, USA). WIP1 inhibitor GSK2830371 (here referred to as WIP1i and used at 0.5 μM unless stated otherwise), olaparib and camptothecin (all Medchemexpress, New York, NJ, USA) and mitomycin C (Santa Cruz Biotechnology, Dallas, TX, USA) were dissolved in DMSO and used at indicated concentrations.

### 4.3. Immunoprecipitation

HEK293 or U2OS-WIP1-KO cells were transfected with GFP-WIP1 plasmid using polyethylenimine. Cells treated as indicated were extracted in lysis buffer [50 mM Tris pH 8.0, 120 mM NaCl, 1% Tween-20, 0.1% NP-40, 1.0% glycerol, 2 mM EDTA, 3 mM EGTA, 10 mM MgCl_2_, complete protease and phosphatase inhibitors (Sigma-Aldrich, St. Louis, MO, USA)] supplemented with benzonase (100 U/mL), briefly sonicated and EtBr (50 μg/mL) was added before centrifugation 30 min 4 °C at 20,000 *g*. GFP-Trap beads (Chromotek, Planegg, Germany) were added to lysate for 1 h before washing 4× with lysis buffer. Alternatively, endogenous WIP1 was immunoprecipitated from U2OS cells by a rabbit affinity-purified antibody generated against human WIP1 and immobilized on protein A/G UltraLink resin (Thermo Fisher Scientific, Waltham, MA, USA). Bound proteins were eluted with 2× loading buffer and analyzed by Western Blotting. 

### 4.4. Immunofluorescence

Cells were seeded on coverslips one day before treatment. Where indicated, cells were pre-extracted 5 min on ice before fixation. Cells were fixed with 4% PFA 15 min RT. Cells were permeabilized using 0.5% Triton-X100 in PBS 5 min and blocked in 1% BSA for 30 min. Where indicated, cells were pulse labeled with EdU 30 min before irradiation and click reaction was performed before primary antibody incubation in 0.1 M Tris pH 8.5, 0.1 M sodium ascorbate, 2 mM CuSO_4_ and 10 μM AlexaFluor 647 azide (Thermo Fisher Scientific, Waltham, MA, USA) for 30 min at RT. Coverslips were incubated with primary antibodies for 2 h in RT, washed 3× in PBS, incubated with secondary antibody for 1 h in RT, washed 3× in PBS, stained with DAPI in PBS for 2 min, washed in dH_2_O and dried before mounting with Vectashield. Images were acquired using Olympus ScanR system equipped with 40×/1.3 UPLFN or 40×/0.9 objective (Olympus, Tokyo, Japan). Nuclei were segmented based on DAPI intensity and foci were identified using Spot detection module. Total and mean intensities of staining per nucleus were determined. FlowJo v10.6.1 software (TreeStar, Ashland, OR, USA) was used to determine of intensity or foci number in particular cell population.

### 4.5. Flow Cytometry

For cell cycle analysis, cells were incubated with EdU 30 min before harvesting by trypsinization and fixation in 70% EtOH. Cells were permeabilized with 0.5% Triton-X100 in PBS 15 min RT, washed in BSA, click reaction was performed before incubation with primary and secondary antibodies. DAPI was added in final concentration of 5 μg/mL in PBS. Percentage of dead cells was determined 3 days after treatment using Hoechst33258 [71]. To analyze repair efficiency by DR-GFP and EJ assays, cells were pretreated 15 min with WIP1i before ISceI transfection using PEI. Percentage of GFP+ cells was determined 3 days after transfection. For traffic light reporter, cells stably expressing Traffic light reporter 1.1 were seeded at 20,000/well one day before transfection with 5 nM siRNA for 2 days. WIP1i was added 15 min before transfection ISceI and pRRL-SFFV-d20GFP.T2A.mTagBFP Donor plasmid (Addgene, Watertown, MA, USA, ID 31485) [48]. Percentage of GFP and mCherry positive cells in BFP positive singlet cells were analyzed three days after ISceI transfection as described [48]. Data were acquired using BD LSRII flow cytometer (Becton Dickinson, Franklin Lakes, NJ, USA) and analyzed using FlowJo (TreeStar, Ashland, OR, USA).

### 4.6. Cell Survival Assays

Cells were seeded 1 day before treatment to 96 well plates at 50–500 cells per well, incubated seven days with treatment before resazurin was added in fresh media at final concentration 30 μg/mL [44]. Fluorescence at excitation wavelength 560 nm and emission 590 nm was measured using Envision plate reader (PerkinElmer, Waltham, MA, USA) after 2 h incubation.

### 4.7. Western Blot

Cells were lysed in 2× lysis buffer, sonicated and protein concentration was determined using BCA protein assay. A total of 20–50 μg lysate was resolved on SDS-PAGE, transferred to nitrocellulose membrane, blocked in 5% milk, incubated with primary and secondary antibodies and developed with ECL.

### 4.8. In Vitro Phosphatase Assay

U2OS-WIP1-KO cells were treated as indicated and nuclear extracts were prepared using hypotonic lysis as previously described [72]. Briefly, cells were washed with PBS, incubated in buffer A (10 mM HEPES-KOH (pH 7.9), 1 mM MgCl_2_, 2.5 mM KCl, 0.5 mM DTT) for 5 min, centrifuged, resuspended in buffer A containing EDTA-free protease and phosphatase inhibitors (Sigma-Aldrich, St. Louis, MO, USA) and dounce homogenized using tight pestle on ice. Nuclei were spun down at 500 *g* 5 min 4 °C and extracted in buffer C (20 mM HEPES pH 7.9, 1.5 mM MgCl_2_, 0.2 mM EDTA, 25% glycerol, 0.5 mM DTT) supplemented with 600 mM KCl on ice for 15 min before centrifugation at max speed 15 min 4 °C. Supernatants were diluted with buffer C to final concentration 150 mM KCl, clarified by centrifugation max speed 15 min 4 °C and stored at −80 °C. For in vitro phosphatase assay 100 μg of nuclear extract was incubated with 250 ng of purified full-length His-WIP1 phosphatase 15 min at 37 °C [30]. Where indicated ATM (KU-55933, 100 μM) or DNA-PK (NU-7441, 50 μM) inhibitor were added into the phosphatase reaction. Reaction was stopped by addition of 4× sample buffer. Protein phosphorylation was determined using phospho-specific antibodies by Western blotting. Alternatively, U2OS cells grown on coverslips were exposed or not to 5 Gy of IR, fixed with 4% paraformaldehyde, permeabilized with 0.5% Triton X-100 and in situ phosphatase assay using purified WIP1-His was performed as described previously [35]. Reaction was stopped by addition of 20 mM NaF and 20 mM β-glycerolphosphate in PBS, samples were stained with γH2AX, BRCA1 S1524 or 53BP1 T543 antibodies and total nuclear intensity was determined using Olympus ScanR.

## Supplementary Materials

The following are available online at https://www.mdpi.com/2073-4409/8/10/1258/s1, Figure S1: WIP1 inhibition impairs HR and increases sensitivity to DNA damage, Figure S2: Loss of WIP1 delays removal of 53BP1 foci in U2OS cells, Figure S3: WIP1 inhibition delays removal of 53BP1 foci in MCF7 cells, Figure S4: WIP1 dephosphorylates BRCA1 and 53BP1 in vitro and in situ, Figure S5: Loss of WIP1 increases BRCA1 phosphorylation at S1524, Figure S6: Loss of WIP1 increases IR-induced phosphorylation of 53BP1 at T543.

## References

[1] Hustedt N., Durocher D. (2016). The control of DNA repair by the cell cycle. Nat. Cell Biol..

[2] Ferretti L., Lafranchi L., Sartori A. (2013). Controlling DNA-end resection: A new task for CDKs. Front. Genet..

[3] Jackson S.P., Bartek J. (2009). The DNA-damage response in human biology and disease. Nature.

[4] Jackson Stephen P., Durocher D. (2013). Regulation of DNA Damage Responses by Ubiquitin and SUMO. Mol. Cell.

[5] Himmels S.-F., Sartori A.A. (2016). Controlling DNA-End Resection: An. Emerging Task for Ubiquitin and SUMO. Front. Genet..

[6] Chen H., Lisby M., Symington L.S. (2013). RPA coordinates DNA end resection and prevents formation of DNA hairpins. Mol. Cell.

[7] Zou L., Elledge S.J. (2003). Sensing DNA Damage Through ATRIP Recognition of RPA-ssDNA Complexes. Science.

[8] Zong D., Adam S., Wang Y., Sasanuma H., Callén E., Murga M., Chaudhuri A.R. (2019). BRCA1 Haploinsufficiency Is Masked by RNF168-Mediated Chromatin Ubiquitylation. Mol. Cell.

[9] Sy S.M.H., Huen M.S.Y., Chen J. (2009). PALB2 is an integral component of the BRCA complex required for homologous recombination repair. Proc. Natl. Acad. Sci. USA.

[10] Zhao W., Steinfeld J.B., Liang F., Chen X., Maranon D.G., Ma C.J., Song X. (2017). BRCA1–BARD1 promotes RAD51-mediated homologous DNA pairing. Nature.

[11] Chapman J.R., Martin R.G., Taylor J., Simon J., Boulton J. (2012). Playing the End Game: DNA Double-Strand Break Repair Pathway Choice. Mol. Cell.

[12] Panier S., Boulton S.J. (2013). Double-strand break repair: 53BP1 comes into focus. Nat. Rev. Mol. Cell Biol..

[13] Sobhian B., Shao G., Lilli D.R., Culhane A.C., Moreau L.A., Xia B., Greenberg R.A. (2007). RAP80 Targets BRCA1 to Specific Ubiquitin Structures at DNA Damage Sites. Science.

[14] Nakamura K., Saredi G., Becker J.R., Foster B.M., Nguyen N.V., Beyer T.E., Chapman J.R. (2019). H4K20me0 recognition by BRCA1–BARD1 directs homologous recombination to sister chromatids. Nat. Cell Biol..

[15] Fradet-Turcotte A., Canny M.D., Escribano-Díaz C., Orthwein A., Leung C.C., Huang H., Durocher D. (2013). 53BP1 is a reader of the DNA-damage-induced H2A Lys 15 ubiquitin mark. Nature.

[16] Pei H., Zhang L., Luo K., Qin Y., Chesi M., Fei F., Lou Z. (2011). MMSET regulates histone H4K20 methylation and 53BP1 accumulation at DNA damage sites. Nature.

[17] Kleiner R.E., Verma P., Molloy K.R., Chait B.T., Kapoor T.M. (2015). Chemical proteomics reveals a γH2AX-53BP1 interaction in the DNA damage response. Nat. Chem. Biol..

[18] Callen E., di Virgilio M., Kruhlak M.J., Nieto-Soler M., Wong N., Chen H.T., Wesemann D.R. (2013). 53BP1 Mediates Productive and Mutagenic DNA Repair through Distinct Phosphoprotein Interactions. Cell.

[19] Chapman J.R., Barral P., Vannier J.B., Borel V., Steger M., Tomas-Loba A., Boulton S.J. (2013). RIF1 Is Essential for 53BP1-Dependent Nonhomologous End Joining and Suppression of DNA Double-Strand Break Resection. Mol. Cell.

[20] Escribano-Díaz C., Orthwein A., Fradet-Turcotte A., Xing M., Young J.T., Tkáč J., Xu D. (2013). A Cell Cycle-Dependent Regulatory Circuit Composed of 53BP1-RIF1 and BRCA1-CtIP Controls DNA Repair Pathway Choice. Mol. Cell.

[21] Isono M., Niimi A., Oike T., Hagiwara Y., Sato H., Sekine R., Petricci E. (2017). BRCA1 Directs the Repair Pathway to Homologous Recombination by Promoting 53BP1 Dephosphorylation. Cell Rep..

[22] Densham R.M., Garvin A.J., Stone H.R., Strachan J., Baldock R.A., Daza-Martin M., Pearl L.H. (2016). Human BRCA1–BARD1 ubiquitin ligase activity counteracts chromatin barriers to DNA resection. Nat. Struct. Mol. Biol..

[23] Lord C.J., Ashworth A. (2012). The DNA damage response and cancer therapy. Nature.

[24] Lord C.J., Ashworth A. (2017). PARP inhibitors: Synthetic lethality in the clinic. Science.

[25] Ibrahim Y.H., García-García C., Serra V., He L., Torres-Lockhart K., Prat A., Rodríguez O. (2012). PI3K Inhibition Impairs BRCA1/2 Expression and Sensitizes BRCA-Proficient Triple-Negative Breast Cancer to PARP Inhibition. Cancer Discov..

[26] Gogola E., Rottenberg S., Jonkers J. (2019). Resistance to PARP Inhibitors: Lessons from Preclinical Models of BRCA-Associated Cancer. Annu. Rev. Cancer Biol..

[27] Karakashev S., Zhu H., Yokoyama Y., Zhao B., Fatkhutdinov N., Kossenkov A.V., Bitler B.G. (2017). BET Bromodomain Inhibition Synergizes with PARP Inhibitor in Epithelial Ovarian Cancer. Cell Rep..

[28] Zhong Q., Hu Z., Li Q., Yi T., Li J., Yang H. (2019). Cyclin D1 silencing impairs DNA double strand break repair, sensitizes BRCA1 wildtype ovarian cancer cells to olaparib. Gynecol. Oncol..

[29] Kurnit K.C., Coleman R.L., Westin S.N. (2018). Using PARP Inhibitors in the Treatment of Patients with Ovarian Cancer. Curr. Treat. Opt. Oncol..

[30] Macůrek L., Lindqvist A., Voets O., Kool J., Vos H.R., Medema R.H. (2010). Wip1 phosphatase is associated with chromatin and dephosphorylates gammaH2AX to promote checkpoint inhibition. Oncogene.

[31] Macurek L., Benada J., Müllers E., Halim V.A., Krejčíková K., Burdová K., Bartek J. (2013). Downregulation of Wip1 phosphatase modulates the cellular threshold of DNA damage signaling in mitosis. Cell Cycle.

[32] Fiscella M., Zhang H., Fan S., Sakaguchi K., Shen S., Mercer W.E., Appella E. (1997). Wip1, a novel human protein phosphatase that is induced in response to ionizing radiation in a p53-dependent manner. Proc. Natl. Acad. Sci. USA.

[33] Lu X., Ma O., Nguyen T.A., Jones S.N., Oren M., Donehower L.A. (2007). The Wip1 Phosphatase Acts as a Gatekeeper in the p53-Mdm2 Autoregulatory Loop. Cancer Cell.

[34] Shreeram S., Demidov O.N., Hee W.K., Yamaguchi H., Onishi N., Kek C., Minami Y. (2006). Wip1 Phosphatase Modulates ATM-Dependent Signaling Pathways. Mol. Cell.

[35] Shreeram S., Demidov O.N., Hee W.K., Yamaguchi H., Onishi N., Kek C., Minami Y. (2017). ATM/Wip1 activities at chromatin control Plk1 re-activation to determine G2 checkpoint duration. EMBO J..

[36] Shaltiel I.A., Aprelia M., Saurin A.T., Chowdhury D., Kops G.J., Voest E.E., Medema R.H. (2014). Distinct phosphatases antagonize the p53 response in different phases of the cell cycle. Proc. Natl. Acad. Sci. USA.

[37] Cha H., Lowe J.M., Li H., Lee J.S., Belova G.I., Bulavin D.V., Fornace A. (2010). JWip1 Directly Dephosphorylates γ-H2AX and Attenuates the DNA Damage Response. Cancer Res..

[38] Bulavin D.V., Demidov O.N., Saito S.I., Kauraniemi P., Phillips C., Amundson S.A., Kallioniemi A. (2002). Amplification of PPM1D in human tumors abrogates p53 tumor-suppressor activity. Nat. Genet..

[39] Tan D.S., Lambros M.B., Rayter S., Natrajan R., Vatcheva R., Gao Q., Fenwick K. (2009). PPM1D Is a Potential Therapeutic Target in Ovarian Clear Cell Carcinomas. Clin. Cancer Res..

[40] Castellino R.C., de Bortoli M., Lu X., Moon S.H., Nguyen T.A., Shepard M.A., Kim J.Y. (2008). Medulloblastomas overexpress the p53-inactivating oncogene WIP1/PPM1D. J. Neurooncol..

[41] Le Guezennec X., Bulavin D.V. (2010). WIP1 phosphatase at the crossroads of cancer and aging. Trends Biochem. Sci..

[42] Gilmartin A.G., Faitg T.H., Richter M., Groy A., Seefeld M.A., Darcy M.G., Minthorn E. (2014). Allosteric Wip1 phosphatase inhibition through flap-subdomain interaction. Nat. Chem. Biol..

[43] Richter M., Dayaram T., Gilmartin A.G., Ganji G., Pemmasani S.K., van der Key H., Kumar R. (2015). WIP1 Phosphatase as a Potential Therapeutic Target in Neuroblastoma. PLoS ONE.

[44] Pechackova S., Burdova K., Benada J., Kleiblova P., Jenikova G., Macurek L. (2016). Inhibition of WIP1 phosphatase sensitizes breast cancer cells to genotoxic stress and to MDM2 antagonist nutlin-3. Oncotarget.

[45] Pecháčková S., Burdová K., Macurek L. (2017). WIP1 phosphatase as pharmacological target in cancer therapy. J. Mol. Med..

[46] Stolte B., Iniguez A.B., Dharia N., Robichaud A., Conway A., Morgan A., Alexe G. (2018). Genome-scale CRISPR-Cas9 screen identifies druggable dependencies in TP53 wild-type Ewing sarcoma. J. Exp. Med..

[47] Moon S.H., Lin L., Zhang X., Nguyen T.A., Darlington Y., Waldman A.S., Donehower L.A. (2010). Wildtype p53-induced phosphatase 1 dephosphorylates histone variant gamma-H2AX and suppresses DNA double strand break repair. J. Biol. Chem..

[48] Certo M.T., Ryu B.Y., Annis J.E., Garibov M., Jarjour J., Rawlings D.J., Scharenberg A.M. (2011). Tracking genome engineering outcome at individual DNA breakpoints. Nat. Methods.

[49] Jazayeri A., Falck J., Lukas C., Bartek J., Smith G.C., Lukas J., Jackson S.P. (2006). ATM-and cell cycle-dependent regulation of ATR in response to DNA double-strand breaks. Nat. Cell Biol..

[50] Gunn A., Stark J.M., Bjergbæk L. (2012). I-SceI-Based Assays to Examine Distinct Repair Outcomes of Mammalian Chromosomal Double Strand Breaks. DNA Repair Protocols.

[51] Saleh-Gohari N., Bryant H.E., Schultz N., Parker K.M., Cassel T.N., Helleday T. (2005). Spontaneous Homologous Recombination Is Induced by Collapsed Replication Forks That Are Caused by Endogenous DNA Single-Strand Breaks. Mol. Cell. Biol..

[52] Noordermeer S.M., Adam S., Setiaputra D., Barazas M., Pettitt S.J., Ling A.K., Annunziato S. (2018). The shieldin complex mediates 53BP1-dependent DNA repair. Nature.

[53] Schmidt C.K., Galanty Y., Sczaniecka-Clift M., Coates J., Jhujh S., Demir M., Jackson S.P. (2015). Systematic E2 screening reveals a UBE2D–RNF138–CtIP axis promoting DNA repair. Nat. Cell Biol..

[54] Tibbetts R.S., Cortez D., Brumbaugh K.M., Scully R., Livingston D., Elledge S.J., Abraham R.T. (2000). Functional interactions between BRCA1 and the checkpoint kinase ATR during genotoxic stress. Genes Dev..

[55] Xu B., O’Donnell A.H., Kim S.T., Kastan M.B. (2002). Phosphorylation of Serine 1387 in Brca1 Is Specifically Required for the Atm-mediated S-Phase Checkpoint after Ionizing Irradiation. Cancer Res..

[56] Scully R., Chen J., Ochs R.L., Keegan K., Hoekstra M., Feunteun J., Livingston D.M. (1997). Dynamic Changes of BRCA1 Subnuclear Location and Phosphorylation State Are Initiated by DNA Damage. Cell.

[57] Kim H.S., Li H., Cevher M., Parmelee A., Fonseca D., Kleiman F.E., Lee S.B. (2006). DNA Damage–Induced BARD1 Phosphorylation Is Critical for the Inhibition of Messenger RNA Processing by BRCA1/BARD1 Complex. Cancer Res..

[58] Filipponi D., Muller J., Emelyanov A., Bulavin D.V. (2013). Wip1 Controls Global Heterochromatin Silencing via ATM/BRCA1-Dependent DNA Methylation. Cancer Cell.

[59] Chowdhury D., Xu X., Zhong X., Ahmed F., Zhong J., Liao J., Lieberman J. (2008). A PP4-phosphatase complex dephosphorylates gamma-H2AX generated during DNA replication. Mol. Cell.

[60] Lee D.H., Goodarzi A.A., Adelmant G.O., Pan Y., Jeggo P.A., Marto J.A., Chowdhury D. (2012). Phosphoproteomic analysis reveals that PP4 dephosphorylates KAP-1 impacting the DNA damage response. EMBO J..

[61] Penning T.D., Zhu G.D., Gong J., Thomas S., Gandhi V.B., Liu X., Fry E.H. (2010). Optimization of Phenyl-Substituted Benzimidazole Carboxamide Poly(ADP-Ribose) Polymerase Inhibitors: Identification of (S)-2-(2-Fluoro-4-(pyrrolidin-2-yl)phenyl)-1H-benzimidazole-4-carboxamide (A-966492), a Highly Potent and Efficacious Inhibitor. J. Med. Chem..

[62] Lindqvist A., de Bruijn M., Macurek L., Brás A., Mensinga A., Bruinsma W., Medema R. (2009). HWip1 confers G2 checkpoint recovery competence by counteracting p53-dependent transcriptional repression. EMBO J..

[63] Lu X., Bocangel D., Nannenga B., Yamaguchi H., Appella E., Donehower L.A. (2004). The p53-Induced Oncogenic Phosphatase PPM1D Interacts with Uracil DNA Glycosylase and Suppresses Base Excision Repair. Mol. Cell.

[64] Nguyen T.A., Slattery S.D., Moon S.H., Darlington Y.F., Lu X., Donehower L.A. (2010). The oncogenic phosphatase WIP1 negatively regulates nucleotide excision repair. DNA Repair.

[65] Linke S.P., Sengupta S., Khabie N., Jeffries B.A., Buchhop S., Miska S., Yang Q. (2003). p53 Interacts with hRAD51 and hRAD54, and Directly Modulates Homologous Recombination. Cancer Res..

[66] Arias-Lopez C., Lazaro-Trueba I., Kerr P., Lord C.J., Dexter T., Iravani M., Silva A. (2006). p53 modulates homologous recombination by transcriptional regulation of the RAD51 gene. EMBO Rep..

[67] Gatz S.A., Wiesmüller L. (2006). p53 in recombination and repair. Cell Death Differ..

[68] Sriraman A., Radovanovic M., Wienken M., Najafova Z., Li Y., Dobbelstein M. (2016). Cooperation of Nutlin-3a and a Wip1 inhibitor to induce p53 activity. Oncotarget.

[69] Chen Z., Wang L., Yao D., Yang T., Cao W.M., Dou J., Zhao Y. (2016). Wip1 inhibitor GSK2830371 inhibits neuroblastoma growth by inducing Chk2/p53-mediated apoptosis. Sci. Rep..

[70] Wu C.E., Esfandiari A., Ho Y.H., Wang N., Mahdi A.K., Aptullahoglu E., Lunec J. (2017). Targeting negative regulation of p53 by MDM2 and WIP1 as a therapeutic strategy in cutaneous melanoma. Br. J. Cancer.

[71] Kasibhatla S., Amarante-Mendes G.P., Finucane D., Brunner T., Bossy-Wetzel E., Green D.R. (2006). Staining of Suspension Cells with Hoechst 33258 to Detect. Apoptosis. Cold Spring Harb. Protoc..

[72] Shiotani B., Zou L. (2009). Single-Stranded DNA Orchestrates an ATM-to-ATR Switch at DNA Breaks. Mol. Cell.

