# Supplementary material for: WIP1 Promotes Homologous Recombination and Modulates Sensitivity to PARP Inhibitors
Source: Cells. 2019 Oct 15;8(10):1258. doi: 10.3390/cells8101258 (PMC6830099; doi:10.3390/cells8101258)
Supplement: Supplementary file 1 [file cells-08-01258-s001.pdf]

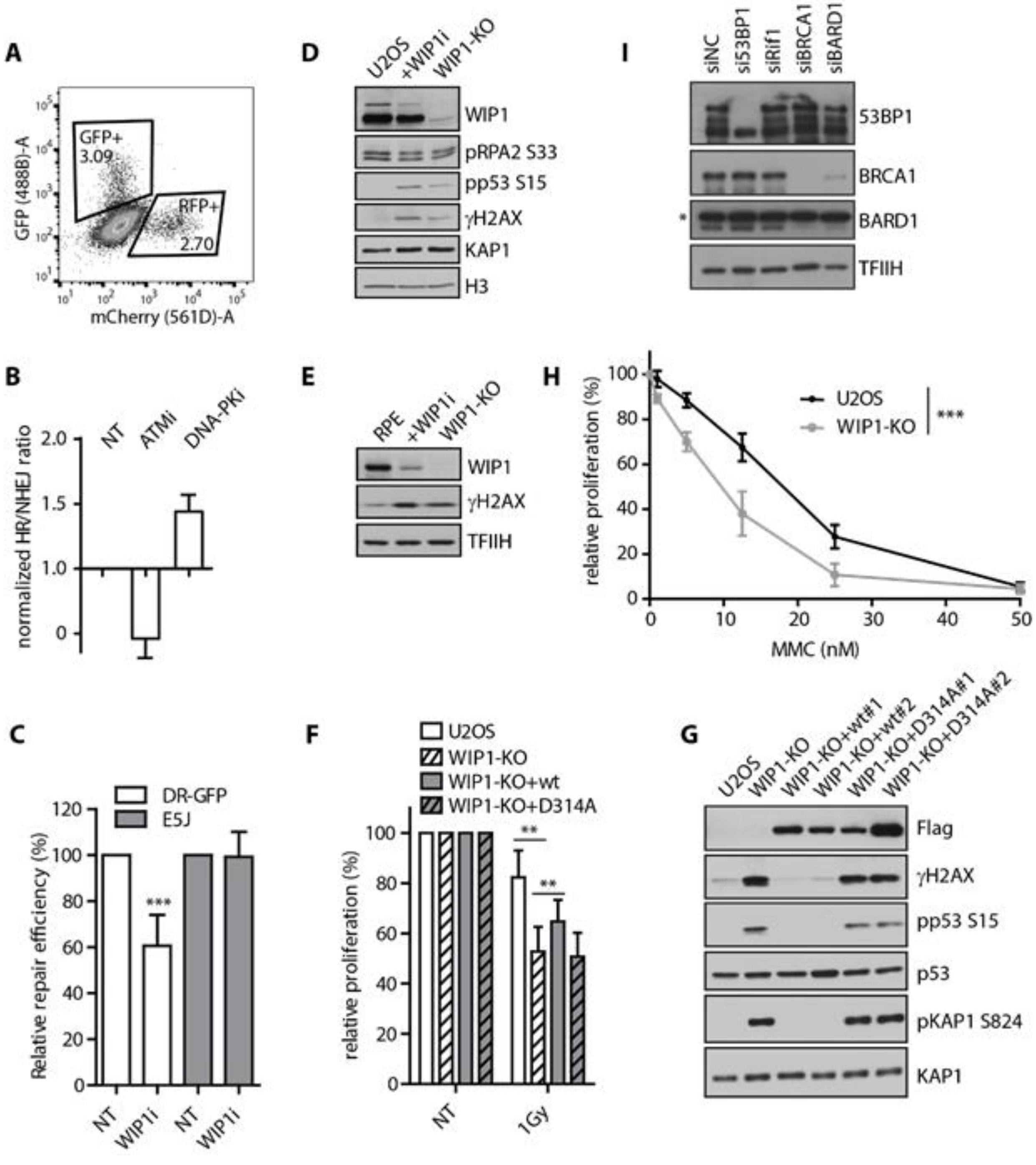

Supplementary Figure 1. WIP1 inhibition impairs HR and increases sensitivity to DNA damage.

A) Representative dot plot of traffic light reporter in U2OS cell line. Dot plot of BFP-positive single cells is showing gating applied to distinguish cells that repaired the break using end-joining (RFP+) or homologous recombination (GFP+). **B)** Cells expressing the traffic light reporter were left non-treated (NT) or incubated with ATM (10 μM) or DNA-PK (5 μM) inhibitors and the ratio between NHEJ and HR was determined by flow cytometry. **C)** Efficiency of repair of double strand break using different reporter cell lines. Efficiency of repair by homologous recombination (DR-GFP) or end-joining (E5J) was evaluated using different reporter cell lines 3 days after transfection of ISceI with or without treatment with 1uM WIP1i. **D)** Western blot analysis of whole cell lysates of U2OS and U2OS-WIP1-KO cells or cells treated with WIP1i for 1h. **E)** Western blot analysis of whole cell lysates of RPE and WIP1 knock-out cells or cells treated with WIP1i for 1h. **F)** Cell survival after irradiation of parental U2OS and U2OS-WIP1-KO cells stably complemented with wild-type or phosphatase-dead (D314A) mutant of WIP1 was evaluated after 7 d using resazurin viability assay. Plotted is mean +/- SD. Statistical significance evaluated by two tailed t-test. **G)** Western blot analysis of whole cell lysates collected 2 h

after irradiation from F. Two independent complemented clones are shown for wt and D314A WIP1. **H)** Sensitivity of cells to MMC. Relative proliferation of U2OS parental and WIP1 knock-out cells was analyzed using resazurin 7 d after treatment with indicated doses of MMC. Plotted is mean and SD, n≥3. Statistical significance evaluated by two-way ANOVA (***P < 0.001). **I)** Western blot analysis of protein depletion after siRNA transfection. Cells were harvested 2 days after transfection with indicated siRNAs and analyzed using Western blotting with indicated antibodies. Asterisk indicates an unspecific band recognized by BARD1 antibody.

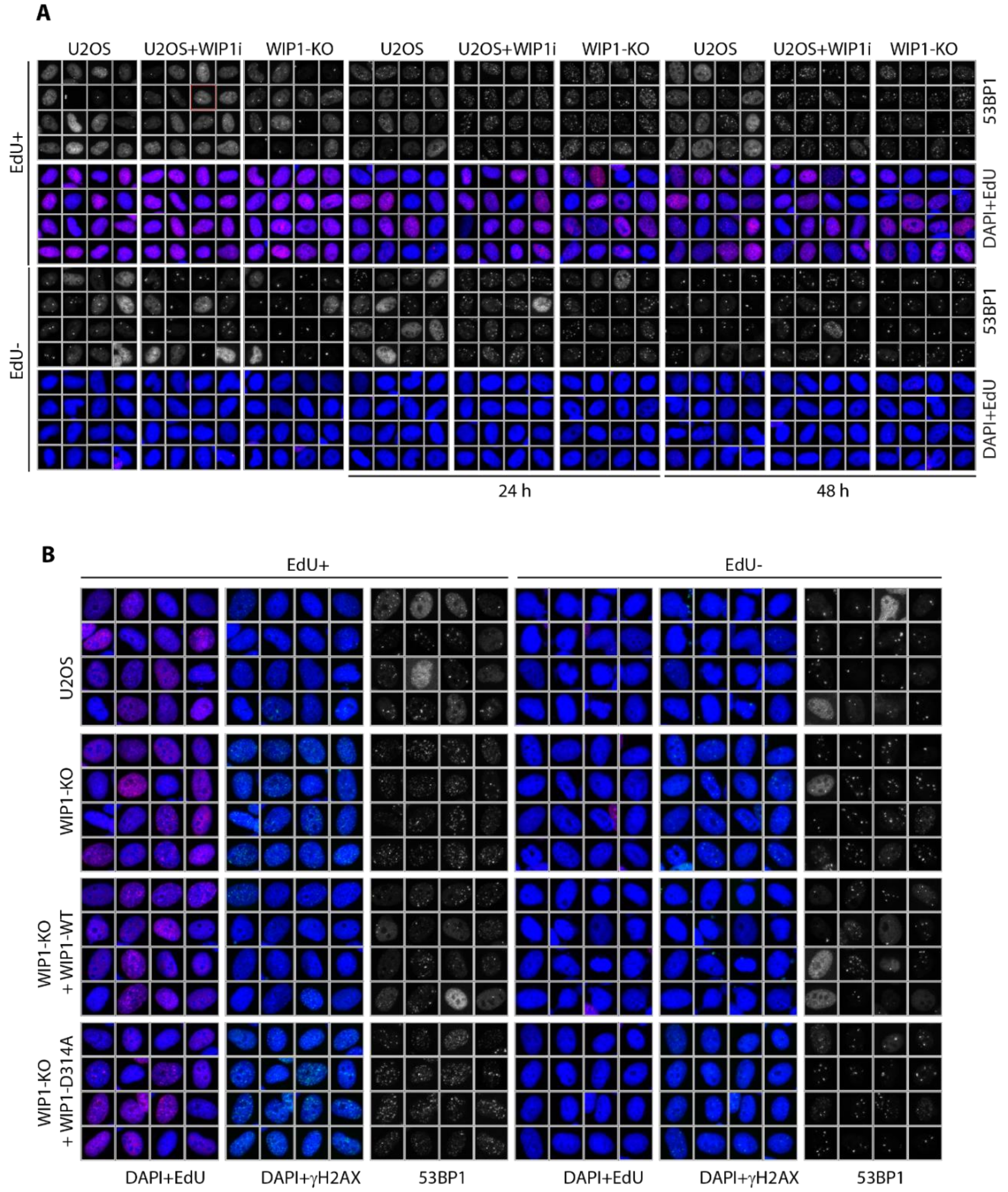

Supplementary figure 2. Loss of WIP1 delays removal of 53BP1 foci in U2OS cells.

A) Representative images from experiments shown in Figure 2 A and 2 B. **B)** Representative images from experiments shown in Figure 2 C and 2 D.

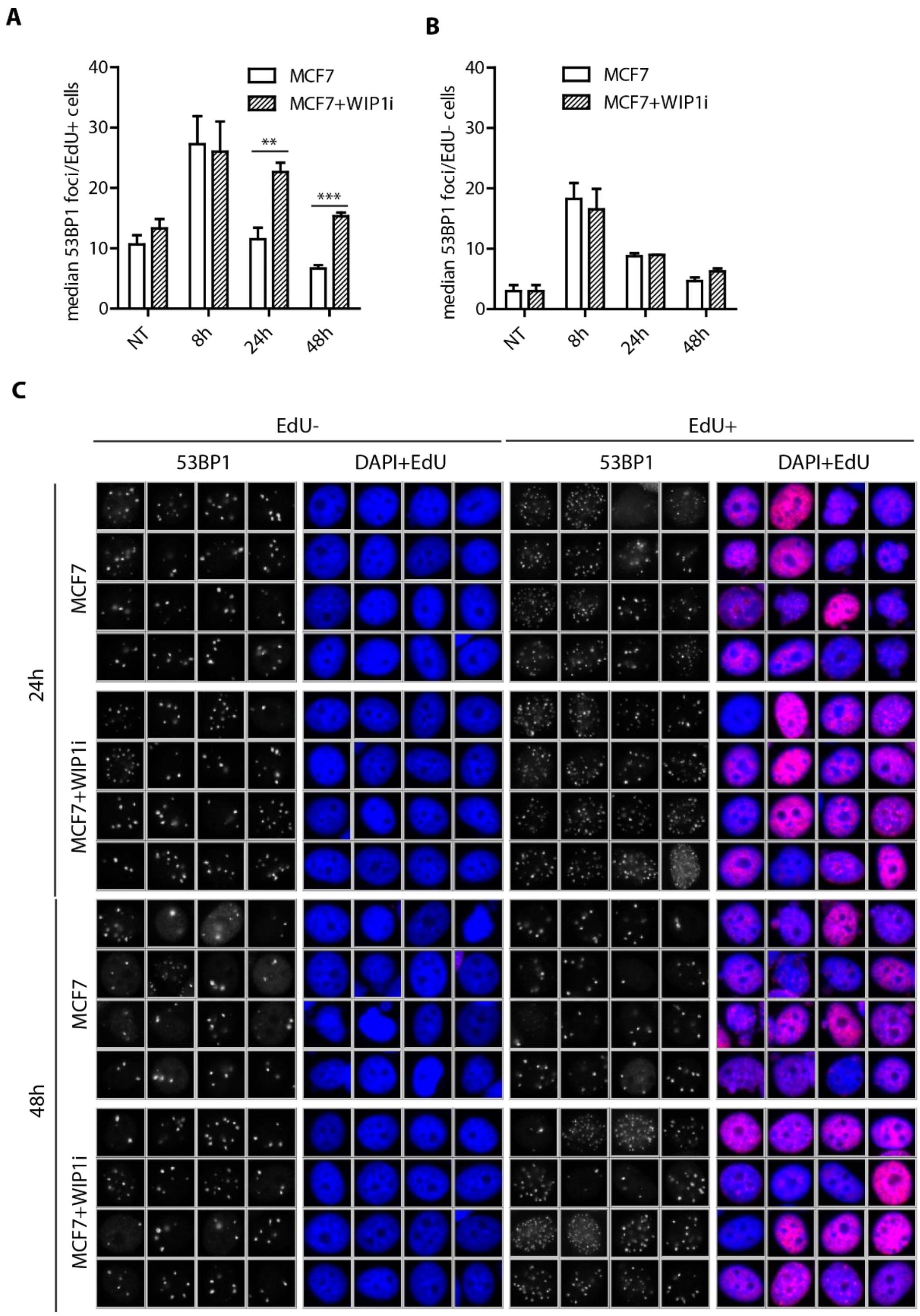

Supplementary figure 3. WIP1 inhibition delays removal of 53BP1 foci in MCF7 cells.

A) Quantification of 53BP1 foci in replicating (EdU+) cells after irradiation. MCF7 cells with or without combined treatment with WIP1i were pulse-labelled with EdU for 30 minutes before irradiation. Cells were fixed after pre-extraction at indicated time-points and stained with γH2AX and 53BP1 antibodies. Click chemistry was used to visualize EdU. Mean of median foci number +/- SD is plotted. Statistical significance evaluated by two tailed t-test. **B)** Quantification of 53BP1 foci in non-replicating (EdU-) cells after irradiation. As in B. **C)** Representative images from A and B.

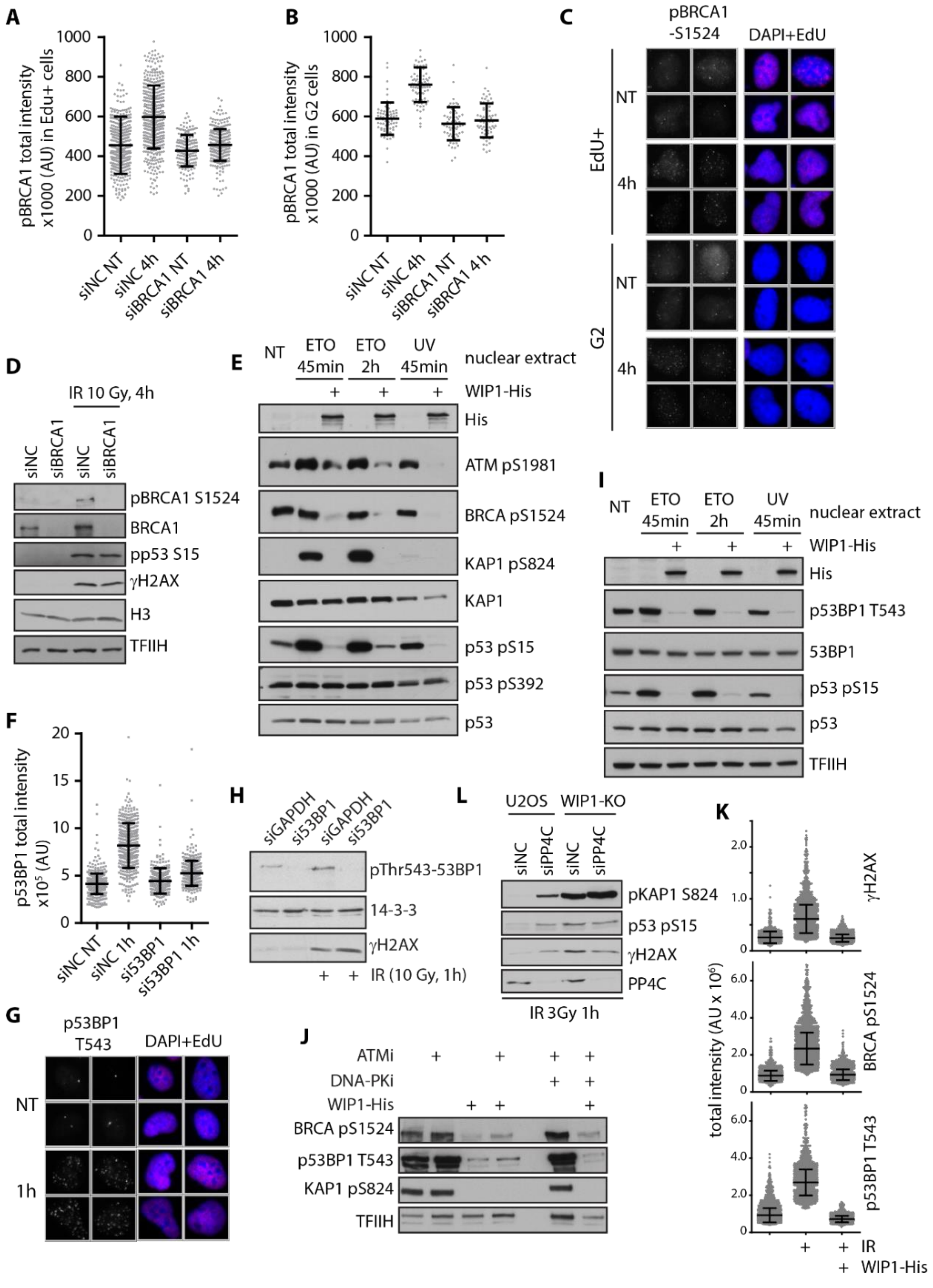

Supplementary figure 4. WIP1 dephosphorylates BRCA1 and 53BP1 *in vitro* and *in situ.*

A) Validation of pBRCA1 S1524 antibody for immunofluorescence in replicating (EdU+) cells. U2OS cells were transfected with NC or BRCA1 siRNA and after 2 days were pulse-labelled with EdU for 30 min and irradiated. After 4 h, cells were fixed after pre-extraction at indicated time-points and stained with pBRCA1 S1524 antibody. Click chemistry was used to visualize EdU. Single cell intensities and mean +/- SD from representative experiment is plotted. **B)** Validation of pBRCA1 S1524 antibody for immunofluorescence in G2 (4n, EdU-) cells. **C)** Representative examples of pBRCA1 S1524 staining from A and B. **D)** Validation of pBRCA1 S1524 antibody

by Western Blotting. U2OS cells were irradiated 2 d after transfection with NC or BRCA1 siRNA and whole cell lysates were analyzes by Western blotting with indicated antibodies. **E)** *In vitro* phosphatase assay using nuclear extracts. Nuclear extracts from untreated U2OS-WIP1-KO cells or after indicated treatments were incubated with purified His-WIP1 at 37°C for 30 min. Reactions were stopped by addition of 4x sample buffer and analyzed by Western Blotting using indicated antibodies. **F)** Validation of p53BP1 T543 antibody for immunofluorescence in replicating (EdU+) cells. U2OS cells were transfected with NC or 53BP1 siRNA and after 2 days were pulse-labelled with EdU for 30 min and irradiated. Cells were fixed after pre-extraction at indicated time-points and stained with p53BP1 T543 antibody. Click chemistry was used to visualize EdU. Single cell intensities and mean +/- SD from representative experiment is plotted. **G)** Representative examples of p53BP1 T543 staining from F. **H)** Validation of p53BP1 T543 antibody for Western Blotting. U2OS cells were transfected with indicated siRNAs and irradiated 2 days later with 10 Gy. Whole cell lysates were analyzed by Western blotting with indicated antibodies. **I)** *In vitro* phosphatase assay using nuclear extracts. Nuclear extracts prepared from untreated U2OS-WIP1-KO cells or after indicated treatments were incubated with purified His-WIP1 at 37°C for 30 min. Reactions were stopped by addition of 4x sample buffer and analyzed by Western Blotting using indicated antibodies. **J)** *In vitro* phosphatase assay using nuclear extracts was performed as in E. Reaction was performed in the presence of DMSO, or ATM (10 μM) and DNA-PK (5 μM) inhibitors. **K)** In situ phosphatase assay. U2OS cells were fixed after exposure to IR, permeabilized, incubated or not with 600 ng of WIP1-His for 30 min at RT and stained for indicated antibodies. Single cell intensities and mean +/- SD from representative experiment is plotted. **L)** Immunoblotting of samples from Figure 4 E.

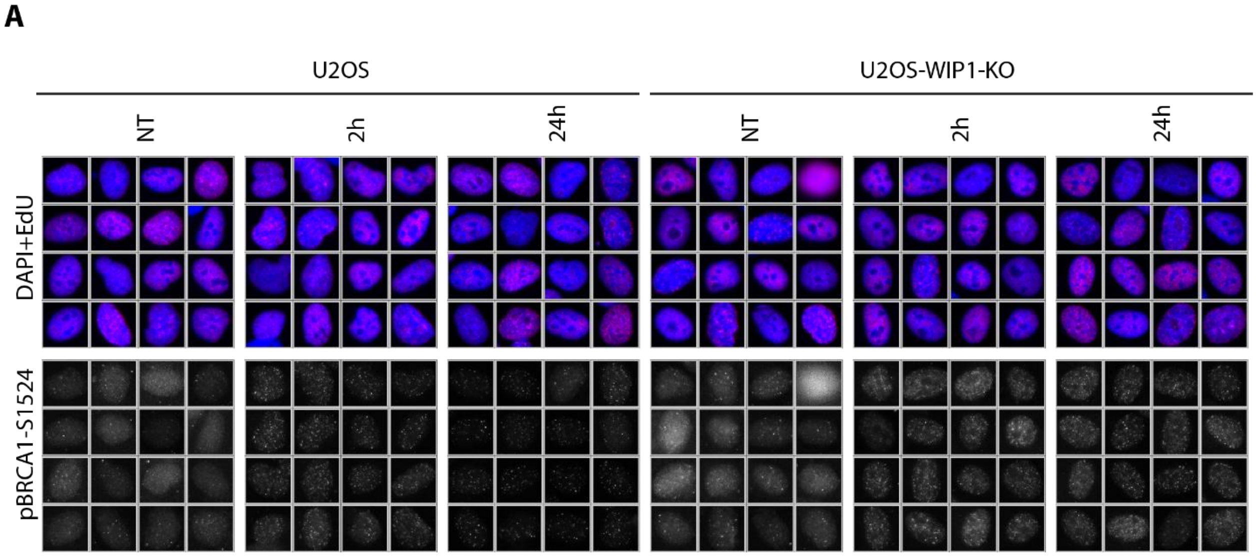

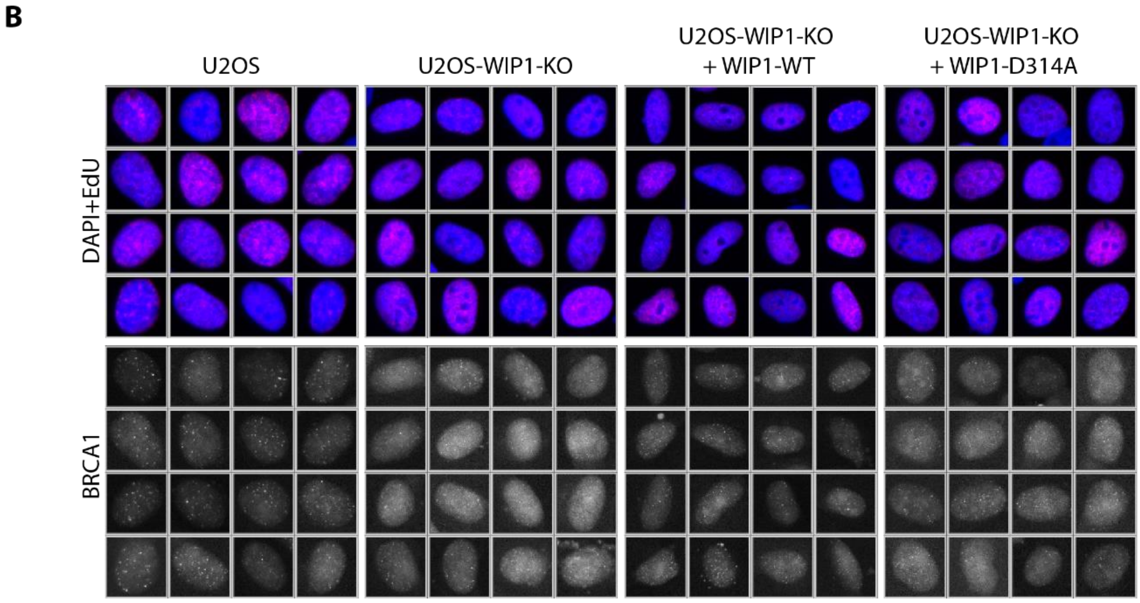

Supplementary figure 5. Loss of WIP1 increases BRCA1 phosphorylation at S1524.

A) Representative images from experiments shown in Figure 3 D. Shown are non-treated cells (NT) and time interval 2 and 24 h after exposure of cells to IR. **B)** Representative images from the complementation assay in Figure 3 G. Shown is the time interval 2 h after exposure of cells to IR.

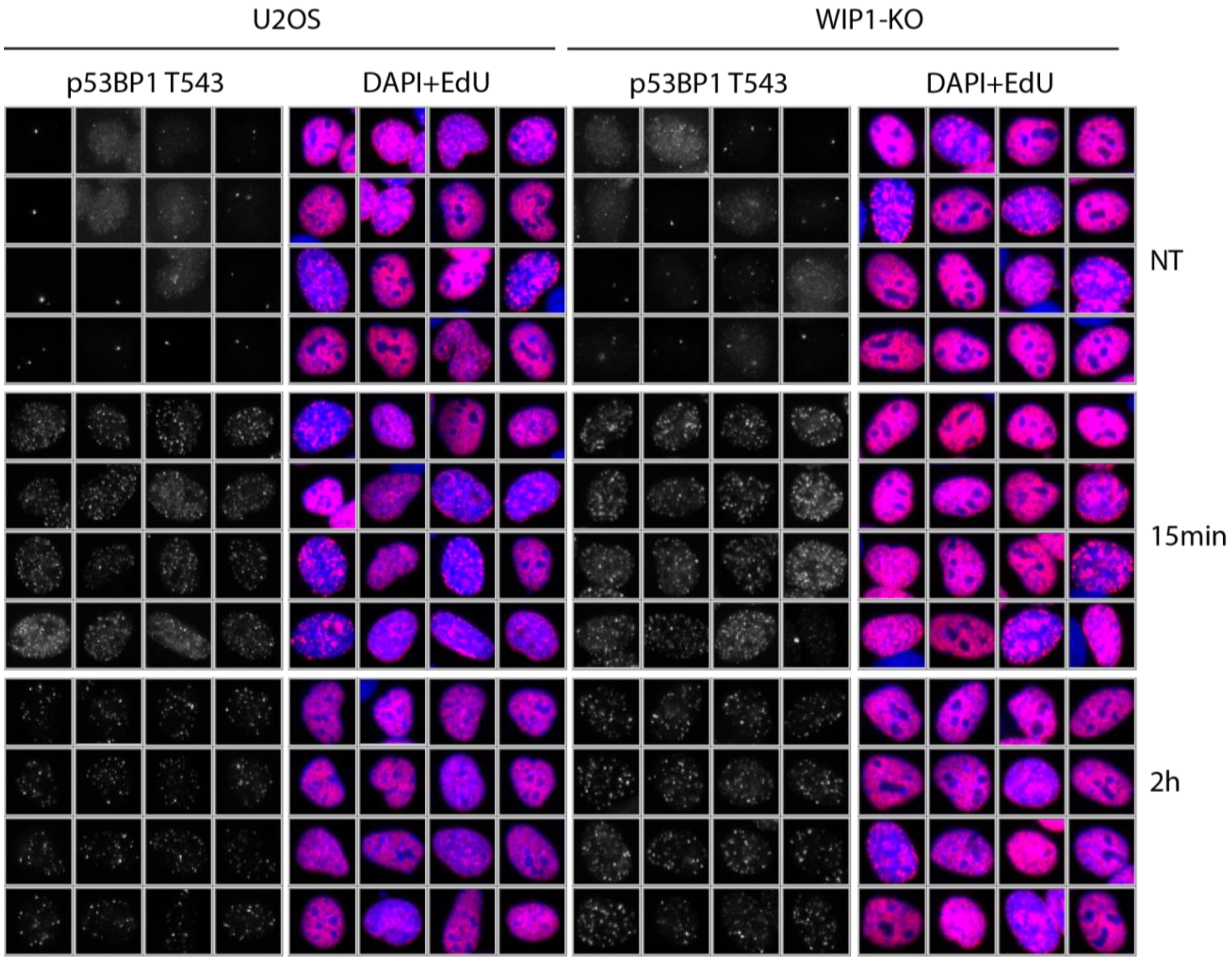

Supplementary Figure 6. Loss of WIP1 increases IR-induced phosphorylation of 53BP1at T543.

Representative images from experiments shown in Figure 4 C

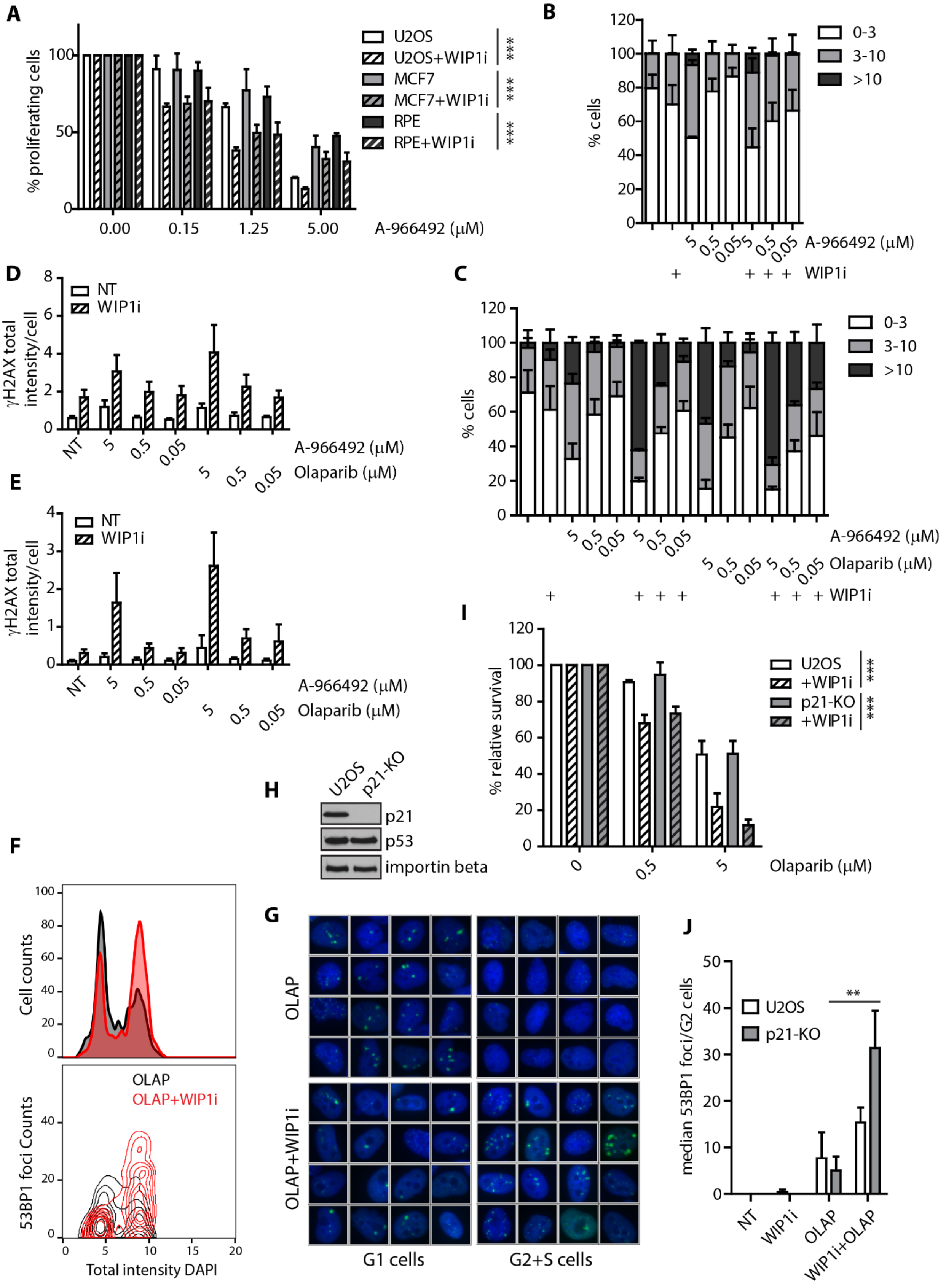

Supplementary Figure 7. Inhibition of WIP1 potentiates DNA damage induced by olaparib.

A) Cell survival of U2OS, MCF7 and RPE cell lines with or without combined treatment with WIP1i was evaluated 7 days after treatment with indicated doses of A-966492 using resazurin viability assay. Plotted is mean +/- SD, n≥3. Statistical significance evaluated by two-way ANOVA. **B)** Quantification of 53BP1 foci number

after treatment with A-966492. U2OS cells were treated with indicated doses of PARPi together with or without WIP1i for 3 days, fixed, stained with 53BP1 antibody and percentages of cells having 0-3, 3-10 and >10 foci were quantified. Bars indicate SD, n≥3. **C)** Quantification of 53BP1 foci number in MCF7 cells after treatment with PARP inhibitors as in B. **D)** Quantification of γH2AX intensity after treatment with PARP inhibitors. U2OS cells were treated with indicated doses of PARP inhibitors together with or without WIP1i for 3 days, fixed, stained with γH2AX antibody and median of the total intensity per cell was quantified. Bars indicate SD, n≥3. **E)** Quantification of γH2AX intensity in MCF7 cells treated as in D. **F)** Quantification of 53BP1 foci throughout cell cycle. Cell cycle profile and contour plot showing number of 53BP1 foci vs. DAPI 3 days after treatment of U2OS cells with olaparib alone or in combination with WIP1i. Representative plot is shown. **G)** Representative images of the cells from panel F. **H)** Western blot analysis of whole cell lysates from parental U2OS cells and U2OS-p21-KO knock-out cell line. **I)** Cell survival of parental U2OS and U2OS-p21-KO cell lines with or without combined treatment with WIP1i was evaluated 7 days after treatment with indicated doses of olaparib using resazurin viability assay. Plotted is mean +/- SD, n≥3. Statistical significance evaluated by two-way ANOVA. **J)** Quantification of 53BP1 foci after treatment with olaparib in G2 cells. U2OS and U2OS-p21-KO cells were pulse labelled with EdU after treatment with olaparib together with or without WIPi for 3 days. Number of 53BP1 foci in G2 (4n, EdU-) cells was quantified. n≥3. Statistical significance evaluated by two tailed t-test.
